# Sex- and age-dependent mitochondrial dysfunction and cognitive impairment in a mouse model of familial hypercholesterolemia

**DOI:** 10.1186/s13293-026-00893-x

**Published:** 2026-04-10

**Authors:** Nathasha Prado-Lopes, Daniel Fagundes, Letícia Tavares, Whitney Santos, Wellinghton Barros, Henrique Moschen, Márcia Mortari, Jair Goulart, Angélica Amato, Jade de Oliveira, Paula Bellozi, Andreza Fabro de Bem

**Affiliations:** 1 Laboratory of Bioenergetics and Metabolism, Department of Genetics and Morphology, Institute of Biology, University of Brasília, Brasília, DF Brazil; 2 Laboratory of Molecular Pharmacology, Department of Pharmacy, Faculty of Health Sciences, University of Brasília, Brasília, DF Brazil; 3 Laboratory of Neuropharmacology, Department of Physiological Sciences, Biology Institute, University of Brasilia, Brasília, DF Brazil; 4 Brazilian National Institute of Science and Technology on Neuroimmunomodulation, Oswaldo Cruz Foundation, Rio de Janeiro, RJ Brazil; 5 Department of Biochemistry, Graduate Program in Biological Sciences: Biochemistry, Institute of Basic Health Sciences (ICBS), Federal University of Rio Grande do Sul (UFRGS), Porto Alegre, Brazil

**Keywords:** Aging, Sexual dimorphism, Behavior, Hypercholesterolemia, Mitochondria

## Abstract

**Background:**

Familial hypercholesterolemia (FH) is a genetic disorder of cholesterol metabolism caused by loss-of-function variants in the low-density lipoprotein receptor (LDLR), resulting in persistently elevated LDL-cholesterol levels in plasma. Although hypercholesterolemia, especially the high levels of LDL, has been linked to an increased risk of dementia, the underlying mechanisms remain unclear. Here, we investigated the effects of sexual dimorphism and aging on metabolic and cognitive functions in a murine model of FH.

**Methods:**

Adult and middle-aged, male and female, C57BL/6 and LDLr^-/-^ mice were used in this study. Behavioral assessments included locomotor activity, spatial memory, and anxiety-like behavior. Plasma lipid profiles were measured, and mitochondrial function in the hippocampus and brown adipose tissue (BAT) was assessed using high-resolution respirometry.

**Results:**

LDLr^-/-^ mice of both sexes exhibited increased cholesterol and triglycerides levels. Male LDLr^-/-^ mice displayed hyperlocomotion in the Open Field (OF) and Elevated Plus Maze (EPM) at both ages, whereas this phenotype emerged in middle-aged female LDLr^-/-^ mice only in OF. Spatial memory impairments were observed in LDLr^-/-^ mice regardless of sex or age. Hippocampal oxygen consumption was reduced in adult males and middle-aged female mice, whereas BAT respiration was impaired in both sexes at middle-aged animals, affecting distinct respiratory parameters. Correlation analyses revealed that elevated cholesterol levels were associated with memory deficits and hyperlocomotion, along with positive correlations between hippocampal and BAT mitochondrial function.

**Conclusions:**

Collectively, these findings demonstrate that FH induces sex- and age-dependent alterations in behavior and mitochondrial metabolism, providing mechanistic insights into the link between FH and neurodegenerative disease risk.

**Graphical abstract:**

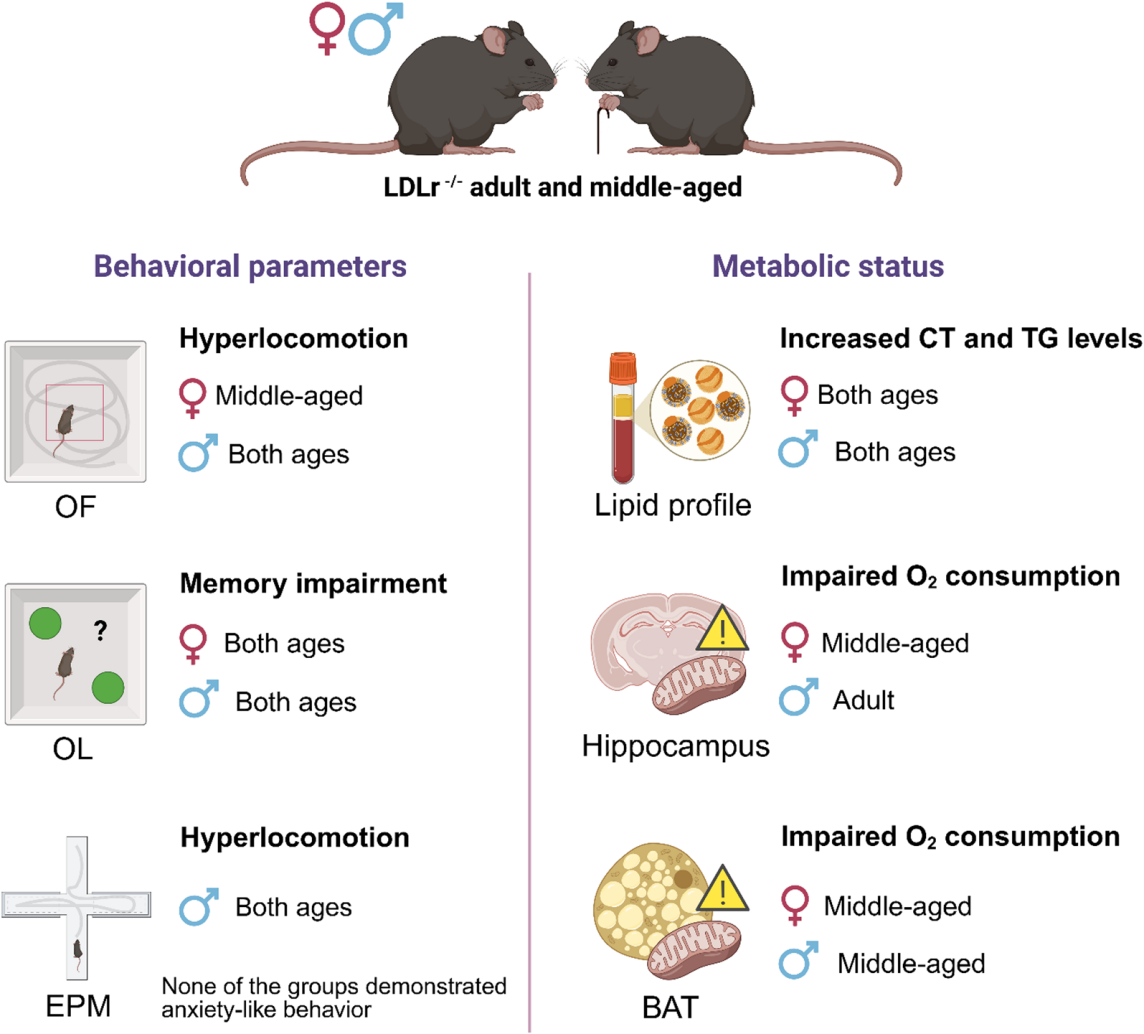

**Supplementary Information:**

The online version contains supplementary material available at 10.1186/s13293-026-00893-x.

## Highlights

- LDLr^-/-^ mice exhibited hyperlocomotion; males were affected at both ages and females at middle-age.
- LDLr^-/-^ mice exhibited spatial memory impairment, independent of sex and age.
- Adult male LDLr^-/-^ mice exhibited higher susceptibility to hippocampal mitochondrial dysfunction; females were affected at middle-age.
- Correlation analyses indicated links between metabolic, mitochondrial, and behavioral outcomes.
- LDLr^-/-^ mice showed age- and sex-dependent differences that may elucidate potential determinant factors in HF outcomes.

**Supplementary Information:**

The online version contains supplementary material available at 10.1186/s13293-026-00893-x.

## Abstract (plain language summary)

Familial hypercholesterolemia (FH) is a genetic condition in which cholesterol levels are high from an early age. Because the body cannot remove cholesterol efficiently, it can build up in the blood and in the arteries over time. FH is best known for increasing the risk of heart disease, but long-term high cholesterol may also affect brain function. In this study, we used a mouse model of FH to understand how high cholesterol affects memory, behavior, and mitochondrial metabolism, and whether these effects differ between males and females as they age. To do this, we studied male and female mice during adulthood and middle age. Mice with FH showed difficulties remembering the location of objects, indicating impaired spatial memory. This memory problem was observed in both sexes. However, other changes differed between males and females. Male mice with FH showed increased activity levels at both ages, while female mice showed this change only at middle age. We also examined how mitochondria function in the hippocampus, a brain region important for memory, and in brown fat, a tissue involved in metabolism. Mitochondrial function was altered in FH, with differences between males and females. Overall, our findings show that FH affects brain function and mitochondrial metabolism differently in males and females. Males were susceptible in adulthood, while females showed effects after middle age, emphasizing the role of sex and age in the long-term impact of high cholesterol.

**Supplementary Information:**

The online version contains supplementary material available at 10.1186/s13293-026-00893-x.

## Introduction

Familial hypercholesterolemia (FH) is one of the most prevalent genetic disorders worldwide, particularly in the heterozygous form [1, 2]. It is mainly caused by mutations in the low-density lipoprotein (LDL) receptor (LDLr) gene and is characterized by chronically elevated LDL-cholesterol levels, resulting in arterial deposition and substantially increased risk of atherosclerotic cardiovascular disease (ASCVD) [3, 4]. Beyond cardiovascular consequences, increasing evidence suggests that FH also compromises brain health. Two aspects are particularly relevant: lifelong exposure to elevated cholesterol levels and LDLr dysfunction [5], which not only regulate cholesterol uptake but also contribute to synaptic function, neuronal plasticity and amyloid-β (Aβ) clearance [6–8]. In this context, the Lancet Commission on Dementia [9] highlights high LDL cholesterol as one of the main modifiable risk factors for dementia. Supporting this link, longitudinal population-based studies have shown that elevated cholesterol levels during midlife are associated with an increased risk of mild cognitive impairment (MCI) and vascular dementia later in life [10, 11]. Evidence from individuals with FH further supports this association, as neuropsychological alterations, including memory deficits, have been reported even in young adults aged 18–40 years [12], suggesting that cholesterol-related cognitive vulnerability may arise earlier than traditionally recognized. More recently, clinical studies in middle-aged individuals with genetically confirmed FH have reported a higher prevalence of cognitive impairment, further indicating that lifelong exposure to elevated LDL cholesterol may contribute to early neurocognitive vulnerability [13]. Consistent with this notion, findings from the ELSA-Brazil cohort revealed nonlinear associations between serum lipid levels and cognitive decline, particularly among individuals younger than 60 years and women [14].

Sexual dimorphism adds further complexity to this scenario. Clinical data indicate a higher prevalence of FH in women, whereas men tend to develop ASCVD earlier and initiate treatment sooner [15–18]. While premenopausal women are relatively protected, menopause attenuates this advantage, raising cardiometabolic and cognitive risks to levels comparable to those observed in men [19, 20]. In parallel, aging is accompanied by systemic metabolic alterations, including insulin resistance, dyslipidemia and chronic inflammation, which indirectly affect brain function and increase the risk of MCI and dementia across the lifespan [21, 22]. As a major risk factor for neurodegenerative diseases, aging entails progressive biological changes that disrupt cholesterol homeostasis and compromise both physical and cognitive functions [23–26]. Therefore, the interplay between sex and aging may critically modulate metabolic and cognitive outcomes in FH.

The LDLr^-/-^ mice, generated by Ishibashi et al. [27], is a well-established model for studying the pathophysiology of FH. Consistent with clinical findings, LDLr^-/-^ mice display elevated LDL-cholesterol levels and a broad spectrum of behavioral alterations, even at young ages, indicating early central nervous system vulnerability. These alterations include deficits in spatial, working and long-term memories [28–32]. Additionally, young and middle-aged LDLr^⁻/⁻^ mice exhibit increased locomotor activity [31, 33], as well as emotional dysregulation, characterized by heightened stress sensitivity and depressive-like behavior [34]. Underlying these cognitive impairments, several cellular and biochemical alterations have been reported in LDLr^⁻/⁻^ mice. These include impaired adult hippocampal neurogenesis, enhanced glial reactivity, blood-brain barrier (BBB) disruption, hippocampal apoptosis, and neuronal and synaptic dysfunctions [29, 34–36]. Among these pathological processes, mitochondrial dysfunction has emerged as a central mechanism, connecting cognitive and neuronal impairments with systemic metabolic alterations.

In the brain, LDLr^-/-^ mice exhibit oxidative stress, impaired respiratory chain function, and reduced coenzyme Q10 (CoQ10) levels, worsened by intracellular cholesterol accumulation driven by mevalonate pathway activation [37–39]. Peripheral tissues are also affected: white adipose tissue (WAT) shows oxidative stress and inflammation, promoting reactive oxygen species (ROS) accumulation, impairing insulin signaling, and contributing to insulin resistance, glucose intolerance, and metabolic dysfunction [40, 41]. WAT from LDLr^-/-^ mice also exhibits macrophage infiltration, increased proinflammatory cytokines, and altered adipokine secretion, establishing a chronic inflammatory state that disrupts energy homeostasis [42, 43]. In contrast, brown adipose tissue (BAT), a thermogenic and mitochondria-rich depot essential for systemic metabolism, regulating thermogenesis, insulin sensitivity, and lipid metabolism [40, 44, 45], remains underexplored in the context of FH.

Notably, most studies have focused on male LDLr^-/-^ mice, leaving the influence of sex largely unresolved. However, sex is a critical determinant of both metabolic regulation and brain function. Given that aging also modulates cholesterol metabolism and hormonal status, FH may exert age- and sex-specific effects on cognition and mitochondrial function. Accordingly, the present study investigated the combined influence of genotype, sex, and age on behavioral and metabolic outcomes in LDLr^-/-^ mice. We assessed cognitive, locomotor and anxiety-like behaviors, as well as mitochondrial function in the hippocampus and BAT, to elucidate mechanisms of vulnerability associated with FH.

## Materials and methods

### Animals

Male and female C57Bl/6 wild-type (WT) and LDLr^-/-^ (B6.129S7 LDLrtm1Her/J) mice, aged 6–8 months (adult group) and 12–14 months (middle-aged group), were originally purchased from the Jackson Laboratory and were bred in our own breeding colony at the University of Brasília. Mice were housed in acrylic cages under controlled conditions, with a filtered air system (Alesco; 3–4 mice/cage) at a controlled temperature (23–25 °C), and 12 h light/dark cycle (lights on at 6 a.m.). They had *ad libitum* access to food and water. All animal procedures complied with the National Institutes of Health (NIH) guidelines for animal care, the Animal Research: Reporting of In Vivo Experiments (ARRIVE) guidelines, and were approved by the Animal Ethics Committee of the University of Brasília (SEI Protocol 23106.075489/2023-95).

### Experimental design

Mice were allocated according on sex, age, and genotype into eight groups of 6–12 animals. Mice weighed between 25­30 g, and were fed a standard rodent diet (SD; 70% carbohydrates, 20% protein, and 10% fat). Behavioral assessments included the open field (OF), object location (OL), and elevated plus maze (EPM) tests. Following this, the animals were anesthetized with ketamine and xylazine via intraperitoneal (i.p.) injection (80:8 mg/kg, respectively) until full analgesia and then were euthanized by cervical dislocation. Blood and tissues, including hippocampi and BAT, were collected immediately after euthanasia.

### Behavioral tasks

For the behavioral tests, the animals were transferred to the testing room at least 1 h before the beginning of the experiments, to allow acclimation. The testing room was maintained at controlled temperature and humidity, mirroring the conditions of their regular housing. The room was also free from extraneous odors, including those from the experimenter (i.e., perfume, deodorant, or lotion were avoided). All behavioral tests were recorded using AnyMaze^®^ software (RRID: SCR_014289; version 7.1 for Windows) and conducted between 7 a.m. and 4 p.m., during the light phase of the animals’ light/dark cycle.

#### Open field (OF)

OF was used to evaluate the spontaneous locomotor and exploratory activities induced by a novel environment. The OF apparatus was a 30 cm length cubic arena with a white background, containing spatial cues on the sidewalls. Animals were placed individually on the center of the apparatus to freely explore the arena for 5 min [46]. The arena was cleaned with ethanol 30% between each animal trial. Total distance traveled and time spent in the inner and peripheral quadrants (inner quadrant was drawn 7.50 cm away from the walls) were evaluated using ANY-maze tracking system (Stoelting Co., IL, USA).

#### Object location test (OL)

The OL test was performed in the same arena used for the OF test to assess spatial reference memory, following the protocol previously described by Assini et al. [47]. During the training session, animals were placed in the arena for 5 min with two identical objects positioned parallel to each other, 5 cm away from the walls. After the training phase, the mice were removed from the arena for 90 min. Following the inter-trial interval, one object remained in the same location (nondisplaced object [ND]), and the other one was relocated to a new position (displaced object [D]). The animals were then reintroduced into the arena and allowed to explore for another 5 min. After each trial, the experimental apparatus was cleaned with 30% ethanol. Exploration time was recorded when the mice sniffed, looked at, touched, or smelled the object at least 1 cm away. A location index (LI) was calculated to evaluate location memory using the formula: LI = TD * 100 / (TD + TND), where TD and TND are the exploration times for the displaced and nondisplaced objects, respectively [48].

#### Elevated plus maze (EPM)

EPM test is currently performed to assess anxiety-like behavior in rodents [49]. The apparatus is elevated 60 cm from the floor, with four arms (18 cm long, 6 cm wide). Two opposite arms are surrounded by walls (6 cm high, enclosed arms), while the other two arms are devoid of enclosing walls (open arms). The four arms are connected by a central area (6 × 6 cm). The animals were individually placed in the central area and allowed to freely explore for 5 min [29]. The apparatus was cleaned with ethanol solution (30% v/v) and dried with paper towels after each trial, to avoid odor impregnation. During the test, the number of entries into each arm and the total distance traveled were recorded using ANY-maze tracking system (Stoelting Co., IL, USA). To normalize the number of entries into the open arms relative to the total number of arm entries, results were expressed as the percentage of entries into the open arms, calculated as follows: 100 * OA/(OA + CA), where OA corresponds to the number of entries into the open arms and CA to the number of entries into the closed arms.

### Lipid profile analysis

Euthanasia was performed at least 72 h after the completion of behavioral testing to ensure that molecular and metabolic measures were not influenced by the acute stress associated with behavioral testing. After euthanasia, approximately 500 µL of whole blood was collected from the animals. The blood was centrifuged at 3000 g for 10 min to obtain serum. Subsequently, cholesterol (Labtest, Cat# 76) and triglyceride (Labtest, Cat# 87) levels were measured in the serum using colorimetric assays, following the manufacturer’s instructions.

### High-resolution respirometry

Oxygen (O_2_) consumption was assessed using high-resolution respirometry (HRR) with an Oroboros 2k Oxygraph (Oroboros Instruments, Innsbruck, Austria) at 37 °C. The oxygraph system is a closed chamber that measures changes in O_2_ concentration. Any variation in O_2_ levels is attributed to the samples, which utilize the substrates or drugs added during the experiment, and consume O_2_, enabling the assessment of specific mitochondrial states [50].

#### Hippocampal respirometry

Hippocampi were collected and homogenized in 300 µL of reaction buffer [RB: 125 mM sucrose, 65 mM KCl, 2 mM KH_2_PO_4_, 2 mM MgCl_2_, 10 mM HEPES, 0.1 mM EGTA, 0.01% bovine serum albumin (BSA)] using a 5 mL glass-teflon homogenizer. The samples (protein ∼0.200 mg/mL) were added to a 2-mL chamber containing RB. Substrates (all purchased from Sigma-Aldrich) were added sequentially to assess O_2_ flux as follows: pyruvate (P; 5mM) and malate (M; 2,5mM) were added to assess complex I activity. For complexes I + II, succinate (S; 10mM) was added. Oxidative phosphorylation (OXPHOS) was evaluated after the addition of 500 µM ADP, and state 4 (leak) was determined following the addition of oligomycin (OMY; 0.1 µg/mL). Uncoupling of the electron transport system (ETS) was induced with titrated carbonyl cyanide 3-chlorophenylhydrazone (CCCP, final concentration 1–3 µM). Complex II activity was assessed by adding 0.5 µM rotenone, and non-mitochondrial residual respiration was measured after the addition of Antimycin A (AA: 1 µM). ATP-linked respiration was calculated as the difference between oxygen consumption rate in OXPHOS and state 4.

#### BAT respirometry

BAT was collected, weighted and cut into 1 mm³ pieces. Around 10 x the volume (µL) of RB corresponding to BAT weight was added to homogenize the tissue into a 5 mL glass-teflon homogenizer. The samples (protein ∼ 0.135 mg/mL) were added to a 2 mL chamber containing RB and 0.1% additional BSA. Initially, PM (5 and 2.5 mM, respectively) were added, followed by guanosine diphosphate (GDP: 1 mM) to evaluate uncoupling protein-1 (UCP-1) activity. Then, CCCP (3–7 µM final concentration) and rotenone (ROT: 0.5 µM) were sequentially added.

### Statistical analysis

All statistical analyses were performed using R (version 4.3), utilizing key packages including tidyverse for data manipulation, car for ANOVA, emmeans for post-hoc comparisons, vegan for multivariate analysis, mediation for causal modeling, and caret for predictive modeling. Prior to analysis, outliers within each of the eight experimental groups were identified for each biological variable using the 1.5x interquartile range (IQR) rule and were excluded from the respective analyses. The assumption of normality of residuals was checked for all parametric tests. The primary statistical method to assess the main effects of sex, age, and phenotype, as well as their interactions, was a three-way analysis of variance (ANOVA). When significant main effects or interactions were detected, simple effects were assessed via pairwise post-hoc comparisons of the estimated marginal means, applying the Holm-Bonferroni method for p-value adjustment. Partial omega-squared (ω^2^_p_) was calculated as an estimate of effect size for the ANOVA results. Graphs were generated using GraphPad Prism 9.0 (RRID: SCR_002798). All data are presented as mean ± SEM unless otherwise stated. A p-value of < 0.05 was considered statistically significant for all tests.

#### Behavioral and metabolic data correlation

To investigate the linear interrelationships among the phenotypic variables, we calculated Pearson’s product-moment correlation coefficients (ρ). The resulting correlation matrix was visualized as a heatmap. To highlight statistically significant associations and facilitate interpretation, only correlation coefficients with an associated p-value of less than 0.05 were numerically displayed within the heatmap cells. Furthermore, we organized the variables a priori into three functional blocks — ‘Metabolic & Behavioral’, ‘Hippocampal Bioenergetics’, and ‘BAT Thermogenesis’.

#### Data preprocessing and dimensionality reduction

Our initial correlation analyses revealed strong collinearity within specific subsets of variables, particularly those related to mitochondrial respiration and locomotor activity. To mitigate the effects of multicollinearity in subsequent statistical models, and to derive robust, composite metrics for these biological domains, we employed Principal Component Analysis (PCA). We conducted three separate PCAs. The first consolidated five hippocampal mitochondrial respiration variables into a single ‘Hippocampal Bioenergetics Score’. The second combined three BAT variables to create a ‘BAT Thermogenic Score’, and the third integrated two locomotor variables into a ‘Locomotor Score’. We confirmed the suitability of the data for this approach using Bartlett’s test of sphericity and the Kaiser-Meyer-Olkin (KMO) measure of sampling adequacy for each component. For each analysis, we retained the first principal component (PC1), as it explained the majority of the shared variance (84.3% for hippocampal, 52.5% for BAT, and 71.8% for locomotor variables).

#### PLS-DA model

We employed a Partial Least Squares Discriminant Analysis (PLS-DA), a supervised machine learning method, to identify the multivariate phenotypic signature capable of discriminating animals by genotype. The predictor set included the metabolic, behavioral, and principal component (PC) scores for bioenergetics; the individual variables comprising the PCs were excluded to prevent multicollinearity. Prior to model training, we autoscored all predictors (mean-centered and scaled to unit variance). We assessed the model’s robustness and performance using a 10-fold cross-validation procedure. The optimal number of latent components for the final model was selected based on the maximization of the Area Under the Receiver Operating Characteristic (ROC) curve. We quantified each variable’s importance in discriminating between genotypes using the Variable Importance in Projection (VIP) scores, where higher values indicate a greater contribution to group separation.

## Results

### Lipid metabolism and BAT thermogenesis are differently affected in male and female LDLr^-/-^ mice

**Fig. 1:**
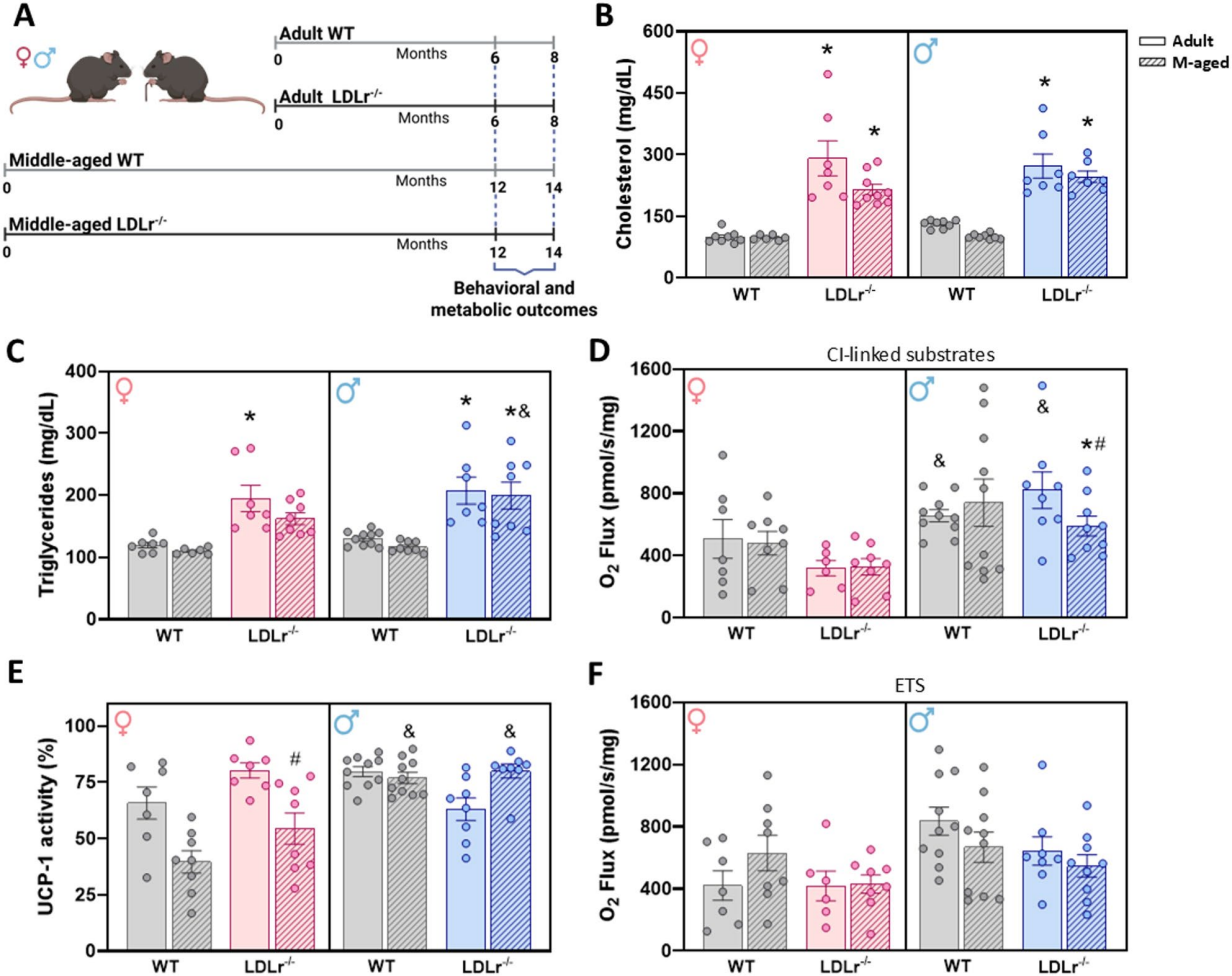
The effect of age, sex, and LDLr genotype on circulating lipid levels and brown adipose tissue’s thermogenic capacity. (**A**) Eight experimental groups: Male and female, adult and middle-aged, Wild-type (WT) and LDLr^⁻/⁻^ mice. Adult mice were 6–8 months old, and middle-aged mice were 12–14 months old. These animals were subjected to behavioral testing, including the OF, OL and EPM tasks. Blood was collected post-euthanasia for lipid profiling: (**B**) Total cholesterol and (**C**) triglyceride levels. HRR was performed to evaluate BAT mitochondrial respiration for (**D**) Complex I-linked substrates (PM), (**E**) UCP-1 activity (GDP), and (**F**) electron transport system (ETS) uncoupling state. *n* = 6–10/group. All data were expressed as mean ± SEM. Statistical analysis was performed using a Three-way ANOVA to include sex, age and genotype variables. *Genotype effect (WT vs. LDLr^-/-^), ^#^Aging effect (WT vs. WT, and LDLr^-/-^ vs. LDLr^-/-^), ^&^Sex effect (Male vs. Female of the same age and genotype), *p* < 0.05

The experimental design included three variables — age (adult vs. middle-aged), genotype (WT vs. LDLr^-/-^), and sex (female vs. male), resulting in eight experimental groups (Fig. 1A). To assess the impact of sex and aging on lipid metabolism in the context of FH, we performed a comprehensive lipid profile analysis. Three-way ANOVA revealed a significant effect of genotype on cholesterol (F(1,51) = 51.57, *p* < 0.001, ω^2^_p_ = 0.68; Fig. 1B) and triglycerides (F(1,51) = 14.91, *p* < 0.001, ω^2^_p_ 0.47; Fig. 1C) levels, as well as a age: genotype interaction on cholesterol levels (F(1,51) = 4.38, *p* = 0.0414, ω^2^_p_ = 0.01). Post-hoc Holm–Sidak test confirmed that both male (both ages: *p* < 0.0001) and female (adult: *p* < 0.0001; middle-aged: *p* = 0.0007) LDLr^⁻/⁻^ mice displayed significantly elevated serum cholesterol compared with age-matched WT controls. For triglycerides levels, LDLr^⁻/⁻^ females showed a significant increase at the adult stage compared with WT controls (*p* = 0.0032), whereas LDLr^-/-^ males displayed elevated levels at both adult (*p* = 0.0012) and middle-aged (*p* = 0.0004) stages. Additionally, middle-aged males LDLr^-/-^ exhibited higher triglyceride levels than females LDLr^-/-^ of the same age (*p* = 0.0422).

To evaluate mitochondrial thermogenic activity in BAT, we measured oxygen consumption associated with complex I (Fig. 1D), the percentage of UCP-linked mitochondrial respiration (Fig. 1E), and the maximal uncoupled respiratory capacity (ETS; Fig. 1F). For complex I, three-way ANOVA revealed a significant effect of sex (F(1,51) = 9.50, *p* = 0.0033, ω^2^_p_ = 0.22), and a sex: age interaction (F(1,51) = 6.95, *p* = 0.0111, ω^2^_p_ = 0.08). Post-hoc analysis showed reduced oxygen consumption in middle-aged LDLr^-/-^ males compared with controls (*p* = 0.0192), as well as an age effect in WT males, with middle-aged animals exhibited lower oxygen consumption than adult group (*p* = 0.0014). Sex differences were also detected in adult WT (*p* = 0.0298) and adult LDLr^⁻/⁻^ mice (*p* = 0.0081), with males exhibiting higher oxygen consumption than females. For UCP1-linked respiration, three-way ANOVA revealed significant effects of sex (F(1,53) = 5.14, *p* = 0.0275, ω^2^_p_ = 0.16), a sex: age: genotype interaction (F(1,53) = 7.23, *p* = 0.0096, ω^2^_p_ = 0.09), and a trend toward a genotype effect (F(1,53) = 3.82, *p* = 0.0559). Post-hoc analysis indicated an age effect in LDLr^⁻/⁻^ females, as middle-aged animals showed reduced UCP-1 activity compared with the adult group (*p* = 0.0085). Additionally, sex differences were observed in middle-aged WT (*p* = 0.0125) and LDLr^⁻/⁻^ (*p* = 0.0097) mice, with males displaying higher UCP-1 activity than females. No significant differences were detected for ETS capacity.

### Ldlr^-/-^ mice exhibit spatial memory impairment regardless of sex, while hyperactivity is more pronounced in males

**Fig. 2:**
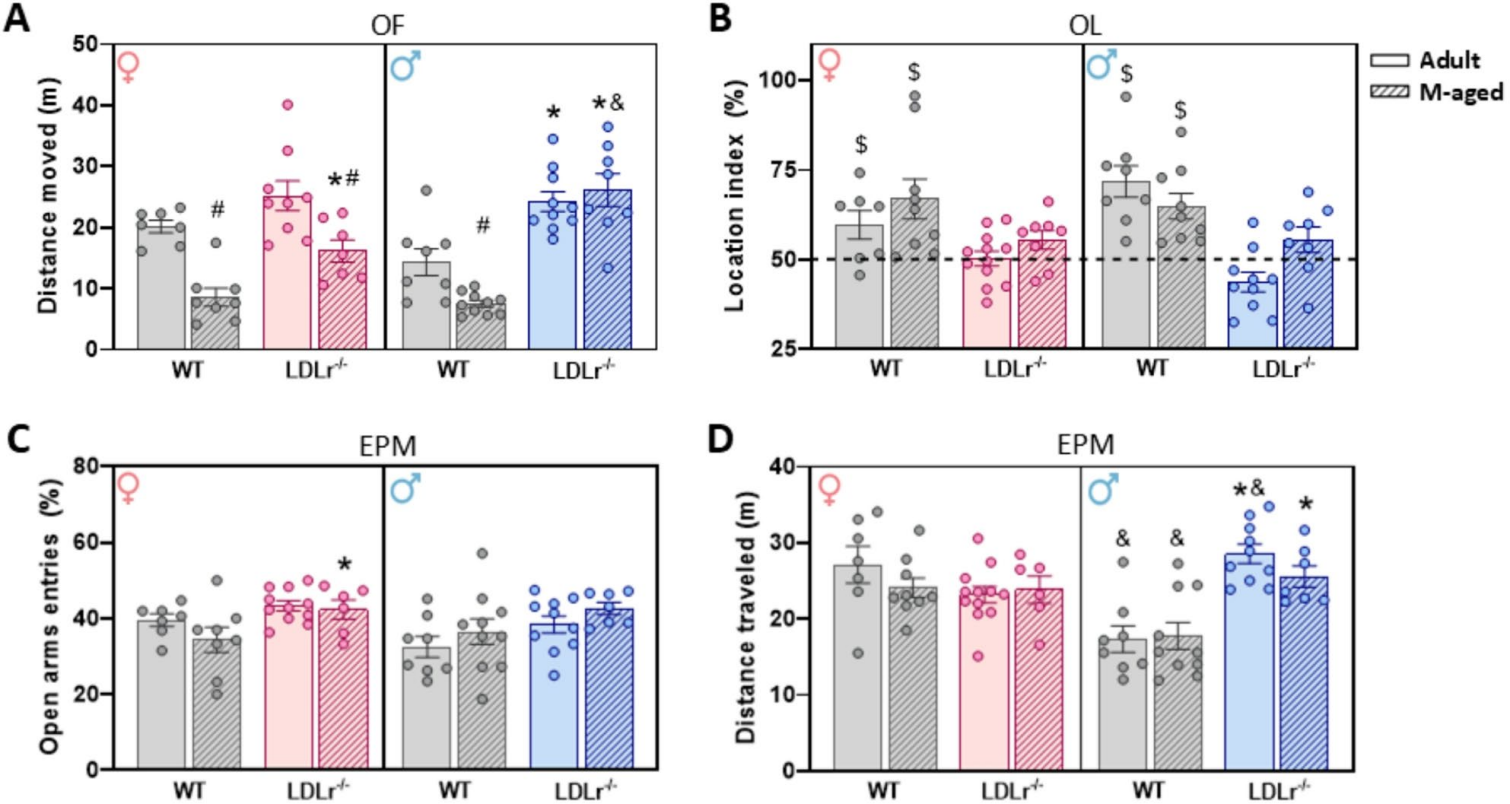
LDLr^-/-^ mice exhibited cognitive impairment and hyperlocomotion. Locomotor performance was evaluated in (**A**) the OF arena for 5 min, and (**B**) the location index was accessed to evaluate cognitive performance. (**C**) Open arms entries (%) and (**D**) distance traveled during the EPM test. *n* = 6–12/group. All data were expressed as mean ± SEM. Statistical analysis was performed using a Three-way ANOVA to include sex, age and genotype variables. *Genotype effect (WT vs. LDLr^-/-^), ^#^Age effect (WT vs. WT, and LDLr^-/-^ vs. LDLr^-/-^), ^&^Sex effect (Male vs. Female of the same age and genotype), *p* < 0.05; ^$^ *p* < 0.05 vs. 50% chance levels

Spontaneous locomotor activity was evaluated in the OF test by measuring the total distance traveled for 5 min (Fig. 2A). Three-way ANOVA revealed significant effects of sex (F(1,60) = 5.54, *p* = 0.0218, ω^2^_p_ = 0.00) and age (F(1,60) = 25.13, *p* < 0.001, ω^2^_p_ = 0.36), as well as a trend toward a genotype effect (F(1,60) = 3.51, *p* = 0.0657, ω^2^_p_ = 0.54). Post hoc analysis showed that LDLr^⁻/⁻^ males exhibited increased locomotion at both ages compared to age-matched WT controls (adult: *p* = 0.0005; middle-aged: *p* < 0.0001), whereas LDLr^⁻/⁻^ females displayed this increase only at middle-age (*p* = 0.0072). An age effect was also detected in WT mice, with middle-aged males (*p* = 0.0247) and females (*p* = 0.0001) traveling shorter distances than their adult counterparts. Similarly, LDLr^⁻/⁻^ females showed reduced locomotion at middle-age compared to adults. In addition, sex differences emerged in middle-aged LDLr^⁻/⁻^ mice, with males traveling greater distances than females of the same age. The percentage of time spent on the periphery of the apparatus (Supplementary Fig. 1A) was also analyzed. Three-way ANOVA revealed significant effects of sex (F(1,56) = 19.64, *p* < 0.001, ω²_p_=0.10), age (F(1,56) = 14.87, *p* < 0.001, ω²_p_=0.05), and a sex: age: genotype interaction (F(1,56) = 14.52, *p* < 0.001, ω²_p_=0.17). Post hoc analysis indicated that LDLr^⁻/⁻^ adult males (*p* = 0.0003) and LDLr^⁻/⁻^ middle-aged females (*p* = 0.0003) spent more time in the periphery compared with their respective WT controls. Moreover, WT females at middle-age spent less time in the periphery than adult WT females (*p* = 0.0027). In addition, a sex effect was detected in adult WT mice, with males spending less time in the periphery than females of the same age (*p* = 0.0004).

Spatial memory was evaluated using the OL test (Fig. 2B). Only WT control groups of both sexes exhibited a location index significantly above the chance level of 50%, while LDLr^⁻/⁻^ mice failed to discriminate the displaced object. Two-way ANOVA revealed a significant effect of age (F(1,61) = 6.50, *p* = 0.0133, ω²_p_=0.16), a sex: age: genotype interaction (F(1,61) = 6.74, *p* = 0.0118, ω²_p_=0.08), and a trend toward a genotype effect (F(1,61) = 3.98, *p* = 0.0505, ω²_p_=0.32). Total exploration time during both the training and test phases did not differ between groups (Supplementary Fig. 5), indicating that differences in the location index reflect memory performance rather than variations in exploratory activity.

Anxiety-like behavior was assessed in the EPM test through the number of entries into open arms (Supplementary Fig. 1B), the percentage of open arm entries (Fig. 2C), and the total distance traveled (Fig. 2D) over 5 min. For the number of entries into open arms, three-way ANOVA revealed significant effects of sex (F(1,58) = 7.59, *p* = 0.0078, ω²_p_=0.00) and a sex: genotype interaction (F(1,58) = 6.67, *p* = 0.0123, ω²_p_=0.08), with a trend for an age effect (F(1,58) = 3.49, *p* = 0.0669, ω²_p_=0.00). Post hoc analysis showed that both adult (*p* = 0.0021) and middle-aged (*p* = 0.0025) LDLr^⁻/⁻^ males displayed increased open arm entries compared to WT controls.

To account for differences in overall locomotor activity, we also calculated the percentage of entries into the open arms relative to the total number of entries. Three-way ANOVA indicated significant effects of age (F(1,61) = 6.44, *p* = 0.0137, ω²_p_=0.00) and a sex: age interaction (F(1,61) = 6.85, *p* = 0.0112, ω²_p_=0.07), as well as a trend toward an age: genotype interaction (F(1,61) = 3.45, *p* = 0.0680, ω²_p_=0.01). Post hoc analysis revealed that middle-aged LDLr^⁻/⁻^ females entered the open arms more frequently than WT females of the same age.

Finally, total distance traveled in the EPM was analyzed to assess locomotor activity within the apparatus. Three-way ANOVA showed significant effects of sex (F(1,55) = 8.34, *p* = 0.0055, ω²_p_=0.00) and a sex: genotype interaction (F(1,55) = 18.72, *p* < 0.001, ω²_p_=0.26). Post hoc analysis demonstrated that LDLr^-/-^ males traveled longer distances than their respective WT counterparts at both ages (adult: *p* < 0.0001; middle-aged: *p* = 0.0012). In addition, sex differences were detected in WT mice, with females traveling longer distances than males at both adulthood (*p* = 0.0442) and middle-age (*p* = 0.0382). A sex effect was also observed in adult LDLr^-/-^ mice, with males covering more distance than females (*p* = 0.0310). To further evaluate whether increased open-arm entries reflected hyperlocomotion rather than altered anxiety-like behavior, we performed a Pearson correlation analysis between the total distance traveled in the OF and open-arm entries in the EPM. Significant positive correlations were observed for the number of entries (*r* = 0.41; Supplementary Fig. 2A) and the percentage of entries (*r* = 0.29; Supplementary Fig. 2B), indicating that mice with higher locomotor activity in the OF also tended to enter the open arms more frequently.

### Sex-specific differences in hippocampal mitochondrial dysfunction in LDLr^-/-^ mice

**Fig. 3:**
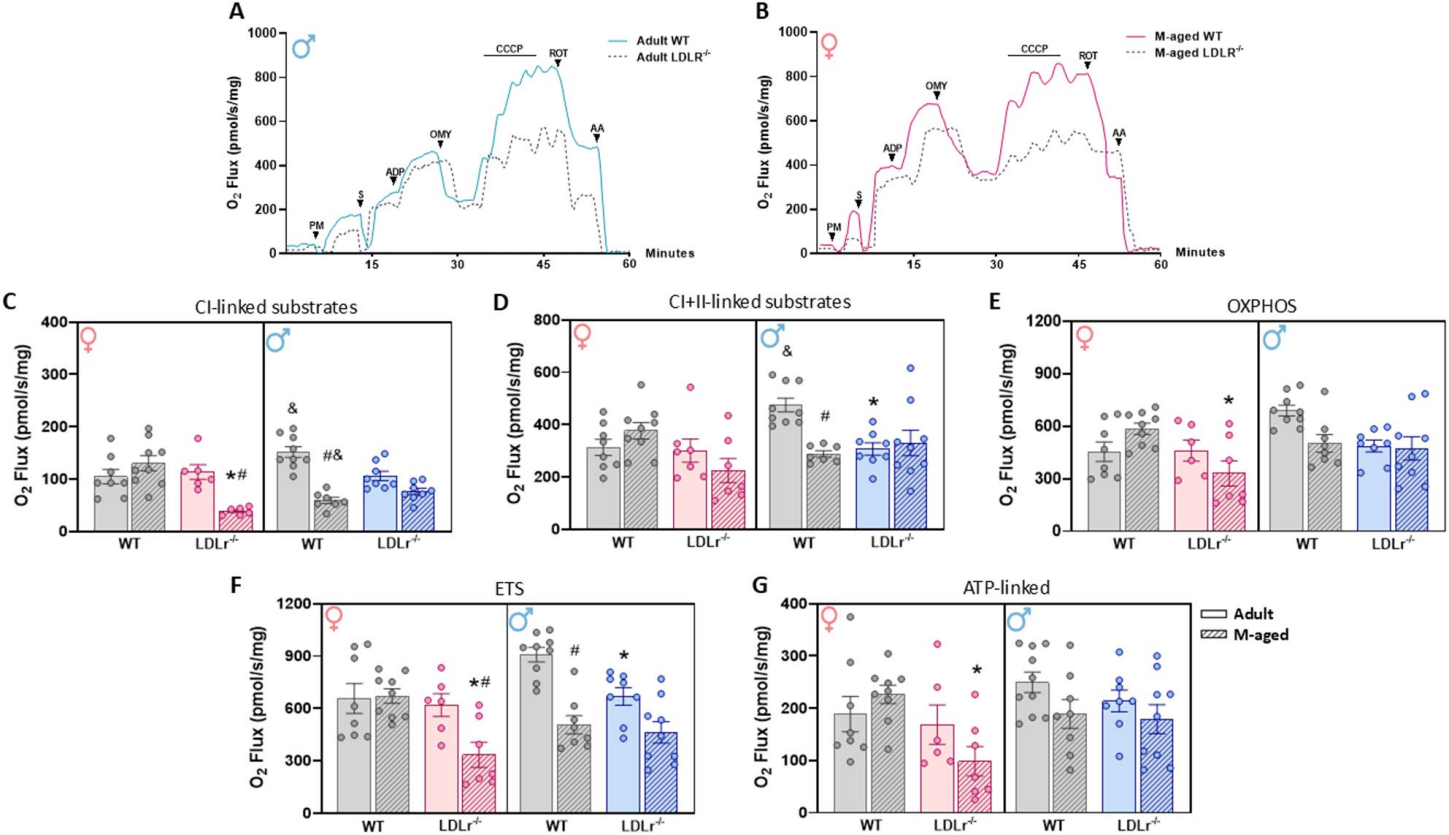
Sex-specific hippocampal mitochondrial impairment in LDLr^-/-^ mice. HRR was performed to evaluate mitochondrial respiratory states: Complex I-linked substrates (PM), Complex I and II-linked substrates (PMS), OXPHOS or state 3 (ADP), state 4 (OMY) and ETS capacity, uncoupled state (CCCP). (**A** and **B**) Representative oxygraphs from young male and aged female mice, comparing control and LDLr^-/-^ groups. (**C**) O_2_ flux (pmol/s) per mg of protein in complex I, and (**D**) Complex I and II, (**E**) OXPHOS and (**F**) ETS capacity. (**G**) ATP-linked oxygen consumption, calculated by the difference between state 3 and state 4. *n* = 6–10/group. All data were expressed as mean ± SEM. Statistical analysis was performed using a Two-way ANOVA to compare differences between same-sex groups. *Genotype effect (WT vs. LDLr), ^#^Age effect (WT vs. WT, and LDLr^-/-^ *v*s. LDLr^-/-^), ^&^Sex effect (Male vs. Female of the same age and genotype), *p* < 0.05

We evaluated hippocampal mitochondrial bioenergetics using HRR to measure oxygen consumption under different stimuli. For complex I–related oxygen consumption (Fig. 3C), three-way ANOVA revealed significant effects of sex (F(1,52) = 8.99, *p* = 0.0042, ω²_p_=0.00), age (F(1,52) = 5.02, *p* = 0.0294, ω²_p_=0.25), and a sex: age: genotype interaction (F(1,52) = 29.44, *p* < 0.001, ω²_p_=0.32), with a trend toward a genotype effect (F(1,52) = 3.91, *p* = 0.0533, ω²_p_=0.09). Post hoc analysis indicated that middle-aged LDLr^⁻/⁻^ females exhibited reduced oxygen consumption compared with WT females (*p* < 0.0001). Age effects were observed in WT males (*p* < 0.0001) and LDLr^⁻/⁻^ females (*p* = 0.0001), with middle-aged animals showing lower mitochondrial activity than adults of the same genotype. Sex differences were also detected in WT mice: adult males displayed higher Complex I–linked respiration than adult females (*p* = 0.0333), whereas middle-aged males exhibited lower activity than females (*p* = 0.0004).

For complex I + II respiration (Fig. 3D), three-way ANOVA revealed significant effects of sex (F(1,53) = 10.59, *p* = 0.0020, ω²_p_=0.02) and a sex: age: genotype interaction (F(1,53) = 10.45, *p* = 0.0021, ω²_p_=0.13). Post hoc analysis indicated reduced mitochondrial respiration in adult LDLr^⁻/⁻^ males compared with WT males (*p* = 0.0143). An age effect was observed in WT males, with middle-aged animals displaying lower oxygen consumption than adults (*p* = 0.0143). Additionally, adult WT males exhibited higher respiration than adult females (*p* = 0.0198).

Regarding OXPHOS capacity (Fig. 3E), three-way ANOVA revealed significant effects of sex (F(1,55) = 8.74, *p* = 0.0046, ω²_p_=0.006) and a sex: age: genotype interaction (F(1,55) = 11.84, *p* = 0.0011, ω²_p_=0.15). Post hoc analysis showed that middle-aged LDLr^⁻/⁻^ females exhibited reduced OXPHOS capacity compared with WT females (*p* = 0.0208). Middle-aged WT males also showed a trend toward reduced capacity compared with adults (*p* = 0.0712), and a similar trend was observed for sex differences in adult WT mice, with males showing higher values than females (*p* = 0.0503).

For ETS capacity (Fig. 3F), three-way ANOVA revealed significant effects of sex (F(1,54) = 7.99, *p* = 0.0066, ω²_p_=0.00) and a sex: age: genotype interaction (F(1,54) = 10.37, *p* = 0.0022, ω²_p_=0.13). Post hoc analysis indicated reduced maximal uncoupled respiration in adult LDLr^⁻/⁻^ males (*p* = 0.0401) and middle-aged LDLr^⁻/⁻^ females (*p* = 0.0019) compared with their WT counterparts. Age effects were also observed in WT males and LDLr^⁻/⁻^ females, as middle-aged animals displayed lower oxygen consumption than adults. Additionally, a trend toward sex differences was observed in adult WT mice (*p* = 0.0527).

Finally, for ATP-linked respiration (Fig. 3G), three-way ANOVA revealed significant effects of sex (F(1,52) = 6.21, *p* = 0.0160, ω²_p_=0.02) and a sex: age: genotype interaction (F(1,52) = 7.58, *p* = 0.0081, ω²_p_=0.10), with a trend toward an age effect (F(1,52) = 3.67, *p* = 0.0608, ω²_p_=0.04). Post hoc analysis demonstrated that middle-aged LDLr^⁻/⁻^ females exhibited reduced ATP-linked respiration compared with WT females (*p* = 0.0119).

### PCA–based functional scores for locomotor, hippocampal, and BAT domains

Following the correlogram analysis (Supplementary Fig. 2), to condense correlated variables into meaningful biological indices, we performed three separated principal component analyses (PCAs). In all analyses, the first principal component (PC1) accounted for the majority of the variance, a finding visually confirmed by the scree plot analysis, where PC1 was the only component across all three domains with an eigenvalue greater than 1.0 (Kaiser’s criterion) (Supplementary Fig. 3).

I. Locomotor Score: the two locomotor variables (OF and EPM) were also condensed into a single component (Bartlett’s χ2 (1) = 12.31, *p* < 0.001; KMO = 0.50). PC1 explained 71.8% of the combined variance, with both distance variables contributing equally (loadings = 0.707). The “Locomotor Score” thus effectively represents an overall measure of locomotor activity.
II. Hippocampal Bioenergetics Score: the five variables related to hippocampal mitochondrial respiration were highly suitable for PCA, as indicated by a significant Bartlett’s test of sphericity (χ2 (10) = 373.57, *p* < 0.001) and a good KMO value of 0.74. PC1, designated the “Hippocampal Bioenergetics Score,” explained 84.3% of the total variance. All five input variables demonstrated strong, negative loadings on this component (loadings from − 0.41 to -0.47), indicating that PC1 represents a robust, integrated measure of overall mitochondrial respiratory function.
III. BAT Thermogenic Score: similarly, the three variables measuring BAT mitochondrial function were appropriate for PCA (Bartlett’s χ2 (3) = 12.2, *p* = 0.007; KMO = 0.62). The resulting PC1, termed the “BAT Thermogenic Score”, accounted for 52.5% of the shared variance. The variables for complex I-linked, maximal, and uncoupled respirations all contributed positively and with similar weights (loadings from 0.56 to 0.59), reflecting a composite index of thermogenic potential.

### Correlational analysis reveals links between metabolic, mitochondrial, and behavioral genotypes

**Fig. 4:**
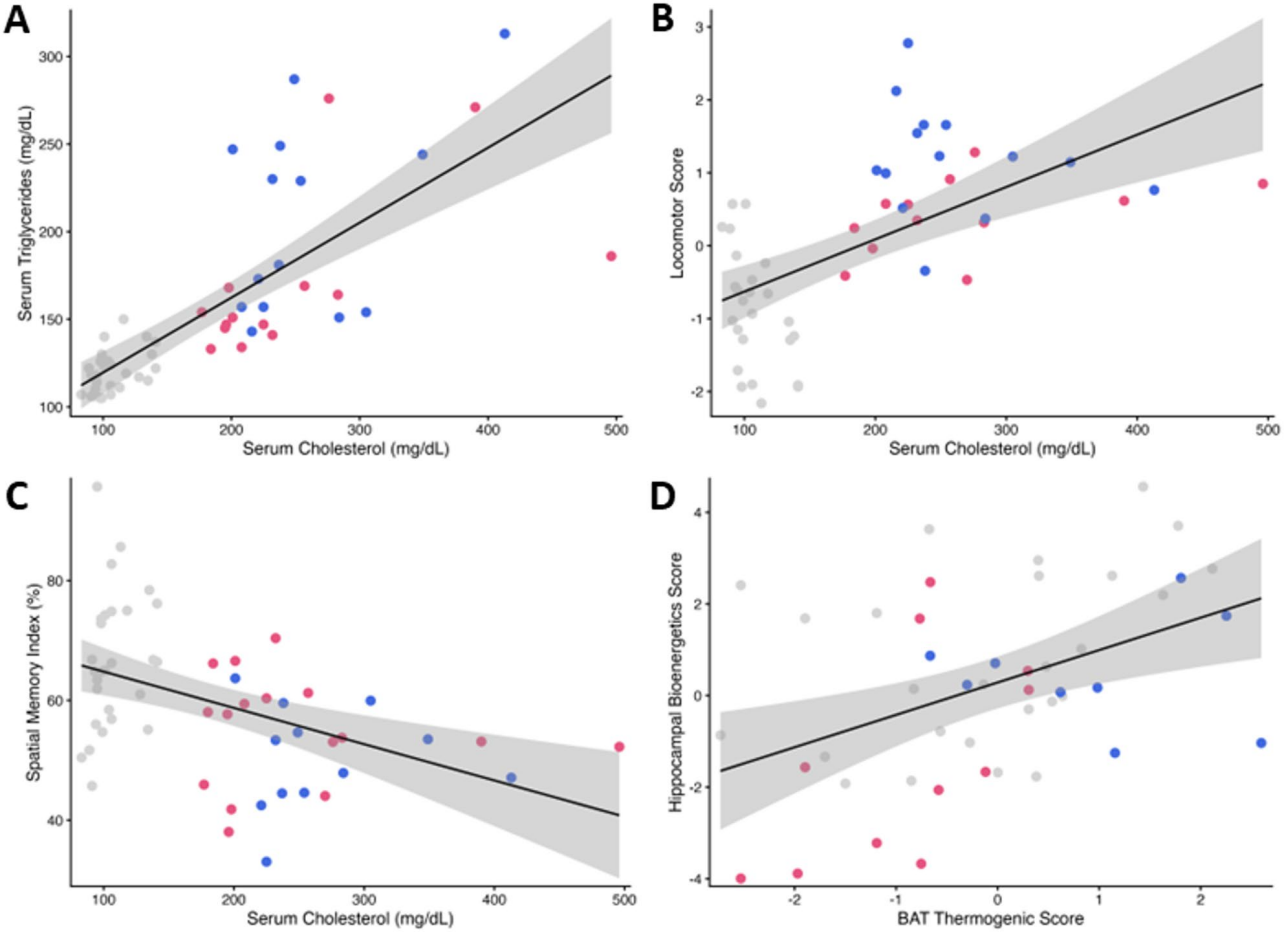
Correlations between key metabolic, behavioral, and bioenergetic parameters. Scatterplots depicting significant linear relationships between select variables. (**A**) A strong positive correlation between serum cholesterol (mg/dL) and triglycerides (mg/dL). (**B**) A positive correlation between serum cholesterol (mg/dL) and the composite locomotor score. (**C**) A moderate negative correlation between serum cholesterol (mg/dL) and the spatial memory index. (**D**) The relationship between the BAT Thermogenic Score and the Hippocampal Bioenergetics Score. In all panels, the solid line represents the best-fit line from a linear regression model, and the shaded area indicates the 95% confidence interval. In the correlation data plots, gray dots represent controls, pink dots represent female LDLr^−/−^ mice, and blue dots represent male LDLr^−/−^ mice. These graphs include animals from both age groups, adult and middle-aged

To elucidate the interrelationships between the observed alterations, we performed a comprehensive Spearman’s correlational analysis (Fig. 4). Critically, metabolic dysregulation was tightly linked to the behavioral genotype: the composite locomotion score was strongly and positively correlated with cholesterol levels (ρ = 0.620, *p* < 0.001). Conversely, cognitive performance was inversely related to metabolic health, with the spatial memory index showing significant negative correlations with serum cholesterol levels (ρ = −0.421, *p* = 0.001). Furthermore, a novel positive correlation was found between overall hippocampal bioenergetic and BAT thermogenic scores (ρ = 0.392, *p* = 0.006), which may reflect an association between central and peripheral bioenergetic impairment.

### Predictive modeling identifies dyslipidemia and hyperactivity as the core genotypic signature

To move beyond identifying group differences and instead determine the core “genotypic signature” of the LDLr^⁻/⁻^ model, we employed a supervised machine learning approach, Partial Least Squares Discriminant Analysis (PLS-DA), to classify animals based on their complete biological profile. Remarkably, the PLS-DA model distinguished LDLr^⁻/⁻^ from control mice with perfect accuracy in cross-validation (ROC-AUC = 1.00), indicating that the measured variables created a highly consistent and separable biological fingerprint for the genotype. An analysis of Variable Importance in Projection (VIP) scores provided a crucial insight into this signature: the most powerful and reliable predictors were serum cholesterol (VIP = 100.00), the composite locomotor score (VIP = 93.55), and serum triglycerides (VIP = 91.33) (Supplementary Fig. 4).

## Discussion

Epidemiological studies over the past decades have consistently shown that high cholesterol is linked to a greater risk of dementia. This connection is even stronger in familial hypercholesterolemia (FH), a condition long known for its cardiovascular complications and now increasingly associated with cognitive problems. In this regard, LDLr^⁻/⁻^ mice are widely used to explore how chronically elevated cholesterol affects tissues that are sensitive to metabolic stress, including the brain, providing an important model for interpreting our findings [27, 51]. In our study, both adult and middle-aged male and female LDLr^⁻/⁻^ mice, fed a standard diet, exhibited impaired cholesterol clearance and significantly elevated plasma cholesterol levels compared to age- and sex-matched WT controls. The increase was approximately two-fold, consistent with the well-characterized hypercholesterolemic phenotype of the model. However, we did not detect sex differences in cholesterol levels at either age. Previous studies have reported varying results: Ishibashi et al. (1993,1994) reported higher cholesterol in female LDLr^⁻/⁻^ mice at 56 days and 6 months [27, 51], while Marsh et al. (1999) observed higher levels in male LDLr^⁻/⁻^ mice at 4 months [52], and Ghosh et al. (2020) described no sex differences at 6 months in LDLr^−/−^ mice on a standard diet [53]. Similarly, Rinninger et al. (2014) reported that both male and female LDLr^⁻/⁻^ mice showed markedly elevated and triglycerides compared to WT [54]. The discrepancies between studies may stem from methodological and physiological variables, including fasting status, diet composition, age, and hormonal influences, all of which can markedly affect lipid metabolism and mask potential sex-related differences.

Conversely, sex- and age-dependent differences in triglyceride (TG) levels were observed in our study. Adult female LDLr^⁻/⁻^ mice displayed elevated TG compared to WT, while male LDLr^⁻/⁻^ mice exhibited increased TG at both ages. While some studies did not observe differences between LDLr^⁻/⁻^ and WT [27, 35], others described pronounced TG elevations in male LDLr^⁻/⁻^ mice, even under standard diet conditions [55, 56]. The Jackson Laboratory also notes that TG increases can be detected in LDLr^⁻/⁻^ mice on chow diet, though they become more evident under high-fat feeding. A similar profile has been reported in LDLr^⁻/⁻^ rats, with increased plasma cholesterol and TG under standard diet [57]. This aligns with the classification of genetic dyslipidemias, in which this form of hypercholesterolemia may include higher TG levels, as LDL particles, while mainly cholesterol-rich, also carry a smaller TG fraction. Given that elevated serum TG reflects atherogenic lipoproteins and adds cardiovascular risk [56], these observations further underscore the systemic metabolic burden caused by LDLr dysfunction, paralleling features seen in FH patients [2].

Thermogenic adipocytes substantially contribute to systemic lipid clearance by accelerating the uptake, oxidation and re-esterification of circulating fatty acids, thereby lowering plasma triglyceride and cholesterol levels [58, 59]. White adipocytes are unilocular with a single large lipid droplet and relatively few elongated mitochondria, whereas brown adipocytes are multilocular and densely packed with mitochondria that are rich in iron and cytochromes, features that underlie BAT’s characteristic coloration and high oxidative capacity [58, 59]. BAT is also highly vascularized and extensively sympathetically innervated, structural attributes that support rapid fuel delivery and heat generation [58]. Mechanistically, BAT thermogenesis is mediated by uncoupling protein 1 (UCP-1), which dissipates the proton motive force across the inner mitochondrial membrane and uncouples electron transport from ATP synthesis, converting chemical energy into heat [60, 61]. In our oximetry analyses, middle-aged male LDLr^⁻/⁻^ mice exhibited reduced oxygen consumption linked to complex I substrates, while middle-aged female LDLr^⁻/⁻^ mice showed a selective reduction in UCP-1–related oxygen consumption, indicating age-dependent declines in thermogenic function in both sexes. We also identified sex-related differences in BAT bioenergetics: adult WT males displayed higher respiration rates linked to complex I substrates compared with adult females, and middle-aged LDLr^⁻/⁻^ males showed higher respiration rates than LDLr^⁻/⁻^ females of the same age. Functionally, such BAT dysfunction is expected to impair thermogenic capacity and lipid clearance, thereby contributing to the exacerbated dyslipidemia observed in LDLr^⁻/⁻^ mice.

These experimental observations are consistent with interventional studies in the LDLr^⁻/⁻^ model: pharmacological activation of thermogenic adipocytes with the selective β3-adrenergic agonist CL316,243 reduced plasma lipids and induced regression of atherosclerotic plaques in LDLr^⁻/⁻^ mice [62]. Conversely, surgical removal of adipose depots, including BAT, aggravated metabolic disturbances in LDLr^⁻/⁻^ mice under high-fat feeding [63], demonstrating the protective, lipid-clearing role of brown and beige fat. More recently, β3-adrenoceptor agonists used clinically, such as mirabegron, were reported to lower plasma triglycerides in LDLr^⁻/⁻^ mice, further supporting the translational relevance of BAT activation for lipid control [59]. Notably, however, most of these studies do not disaggregate outcomes by sex, and evidence specifically addressing UCP-1 activity in female LDLr^⁻/⁻^ mice remains limited.

Beyond systemic metabolism, the LDLr^⁻/⁻^ mice has also been used to study neurocognitive function, including behavioral outcomes and brain cellular homeostasis [32]. In the OF test, we found that adult male LDLr^⁻/⁻^ mice exhibited hyperlocomotion at both ages, traveling significantly greater distances than WT males (Fig. 2A), whereas female LDLr^⁻/⁻^ mice displayed hyperlocomotion only at the middle-age. Aging also influenced female behavior, with middle-aged females showing reduced locomotion compared to adults of the same genotype. These findings extend prior studies that reported hyperlocomotion in male LDLr^⁻/⁻^ mice even under standard diet [31, 33, 64], but which did not include females or consider age. While total distance traveled in the OF is often used as a measure of general activity, increased locomotion in a novel environment may also relate to exploratory drive, novelty‑induced responses or traits similar to impulsivity or disinhibition. For example, rodent models of hyperactivity, including spontaneously hypertensive strains, show elevated OF activity as part of broader behavioral phenotypes relevant to attention‑deficit/hyperactivity disorder (ADHD), rather than reflecting motor activity alone [65, 66]. Similarly, genetic models with novelty-induced locomotion exhibit changes in mesolimbic dopaminergic function linked to exploratory behavior [67]. Similarly, recent work has shown that adult female LDLr^⁻/⁻^ mice display hyperlocomotion accompanied by reduced anxiety-like behavior and increased sociability, a phenotype associated with decreased catechol-O-methyltransferase (COMT) expression in the prefrontal cortex, which suggests a disruption in corticolimbic dopaminergic regulation [68].

In the object location (OL) task, both male and female LDLr^⁻/⁻^ mice, independent of age, failed to discriminate the displaced object, unlike WT controls, that explored the relocated object more than 50% of the time. Spatial memory impairment in OL and cognitive decline has been previously reported in male LDLr^⁻/⁻^ mice, even when fed a standard diet [29, 31, 69, 70]. In the present study we demonstrated that female exhibited this memory impairment like males. Furthermore, LDLr^⁻/⁻^ mice have been shown to be particularly susceptible to cognitive disturbances triggered by various stimuli that induce memory deficits, such as intracerebroventricular Aβ₁–₄₀ injection, a peptide implicated in Alzheimer’s disease pathophysiology. These mice display impairments in both spatial and working memory even in the absence of exogenous Aβ, as reflected by poor performance in the object location and spontaneous alternation tasks. In addition, only LDLr^⁻/⁻^ mice injected with Aβ₁–₄₀ exhibited impaired experience-dependent avoidance behavior in the EPM retest, a form of learning in which prior exposure to the apparatus normally promotes increased open-arm avoidance [29].

These findings align with recent human studies showing that FH contributes to cellular dysfunction in the brain and cognitive decline. For instance, a clinical study comparing Heterozygous FH (HeFH) patients with age-matched controls with no history of cognitive-affecting disorders over 50 years old reported that 21.3% of HeFH patients exhibited MCI, compared with only 2.9% of controls, indicating a significant difference. The authors proposed that early exposure to high cholesterol levels or LDLr dysfunction may constitute a risk factor for MCI [5]. Similarly, another study found that HeFH patients aged 18–40 performed worse on tasks assessing verbal memory and executive function compared with controls, and impairments in executive function correlated with higher serum LDL-C levels in the HeFH group [12].

Considering other behavioral assessments, we employed the EPM to investigate the impact of FH on anxiety-like behavior, particularly given the lack of literature on male and female LDLr^⁻/⁻^ mice in the context of aging and sexual dimorphism. In this study, LDLr^⁻/⁻^ males exhibited a higher number of open-arm entries in the EPM at both ages compared with WT controls, whereas middle-aged LDLr^⁻/⁻^ females also showed increased percentage of open-arm entries relative to WT females. Supporting this interpretation, we observed a significant positive correlation between total distance traveled in the OF and the number of open-arm entries in the EPM (Supplementary Fig. 2), indicating that animals exhibiting higher locomotor activity in the OF also tended to enter the open arms more frequently. Conversely, a recent study reported reduced anxiety-like behavior in adult LDLr^⁻/⁻^ mice of both sexes in the EPM test, characterized by increased open-arm exploration [68]. However, the interpretation of EPM parameters must consider the potential influence of locomotor activity, as increases in open-arm entries may reflect heightened exploration or hyperactivity rather than reduced anxiety-like behavior [49, 71]. Indeed, locomotor activity is a known confounding factor in EPM performance; therefore, complementary measures are often required to distinguish anxiolytic-like effects from changes in general activity [72, 73]. Although middle-aged LDLr^⁻/⁻^ females displayed increased locomotor activity in the OF, they did not exhibit a corresponding increase in locomotion within the EPM, nor an increase in the total number of open-arm entries. To date, no studies have specifically examined EPM performance in the LDLr^⁻/⁻^ model. While previous study has reported depressive-like behavior in these animals [34], similar findings in C57BL/6 mice have shown that a high-cholesterol diet promotes both depressive- and anxiety-like behaviors [74], suggesting a potential link between cholesterol dysregulation and emotional alterations.

Given that such emotional alterations often coexist with cognitive deficits, attention has increasingly turned to the hippocampus, a region essential for learning, memory, and mood regulation [75]. Previous studies indicate that FH can impact brain regions involved in cognitive processes, such as the hippocampus [34] and the prefrontal cortex [31], independent of dietary interventions. To investigate the potential mechanisms underlying the cognitive and behavioral impairments observed in LDLr^⁻/⁻^ mice, we evaluated hippocampal mitochondrial function. Interestingly, our findings revealed both sex- and age-dependent alterations in hippocampal bioenergetics in LDLr^⁻/⁻^ mice. Middle-aged LDLr^⁻/⁻^ females exhibited a marked impairment in hippocampal mitochondrial function, characterized by a reduction in oxygen consumption linked to complex I substrates, OXPHOS, ETS, and ATP-linked respiration. In contrast, adult LDLr^⁻/⁻^ males showed reduced oxygen consumption only when both complex I + II substrates were provided, along with a decline in ETS capacity. This pronounced hippocampal bioenergetic failure in middle-aged females likely reflects a metabolic crisis at the neuronal level, with reduced ATP generation compromising processes essential for synaptic maintenance and plasticity. Given that neuronal ATP is predominantly consumed to sustain synapse formation and activity, key determinants of cognitive performance [67], these data suggest that impaired mitochondrial energy metabolism may directly contribute to the cognitive and behavioral deficits observed in female LDLr^⁻/⁻^ mice at midlife. Collectively, these results highlight significant sex-related differences in hippocampal mitochondrial parameters, reinforcing the concept that mitochondrial bioenergetics are inherently sex-dependent and that aging acts as a critical modifier of the neurobiological consequences of LDLr deficiency on CNS function and behavior. Importantly, these metabolic disturbances are consistent with previous reports describing cognitive and behavioral dysfunctions in LDLr^⁻/⁻^ mice, particularly in males. This model has been shown to present impaired synaptic plasticity and hippocampal neurogenesis, resulting in deficits in spatial and working memory, as well as depressive-like behavior [24, 68]. However, most previous studies have focused primarily on males, leaving the impact of sex and age largely unexplored. Our findings extend these observations by revealing that female LDLr^⁻/⁻^ mice are also vulnerable, exhibiting a distinct bioenergetic profile that may underlie their cognitive and affective alterations.

It is difficult to determine the causal hierarchy of the alterations that ultimately impact mitochondrial function in the LDLr^−/−^ brain. One potential contributor is reduced neuronal uptake of cholesterol resulting from LDL receptor deficiency, which may disrupt membrane composition, cellular signaling, and neuronal survival [37, 76]. However, this mechanism likely occurs in conjunction with multiple cellular and functional alterations previously reported in this model. For instance, LDLr^−/−^ mice exhibit increased BBB permeability, astrogliosis, and hippocampal neuronal loss [29, 77], as well as oxidative stress characterized by increased ROS production and impaired antioxidant defenses [28, 31, 35], alterations that may contribute to mitochondrial dysfunction and cognitive impairment in this model. These conditions can collectively impair mitochondrial function and neuronal bioenergetics. Given the interplay among these processes, establishing direct cause–effect relationships remain challenging. Further mechanistic studies will be required to disentangle how LDL receptor dysfunction, cholesterol metabolism, and neurovascular alterations converge to influence mitochondrial function and neuronal energy homeostasis in FH.

In contrast, LDLr^⁻/⁻^ females exhibited a delayed onset of hippocampal mitochondrial dysfunction, which emerged only at middle age. This sex-specific pattern may arise from a combination of biological mechanisms, including hormonal influences and broader metabolic and mitochondrial differences between males and females. The progressive loss of estrogen-mediated protection may also contribute to this effect, as estrogens enhance mitochondrial efficiency and reduce oxidative damage; however, hormonal levels were not measured in the present study. Human evidence supports sex-specific interactions between lipid metabolism and cognition. In a cohort of Chinese adults over 45 years old, elevated total and LDL cholesterol were associated with cognitive decline in women but not in men [78]. Experimental studies further suggest that disruption of estrogen signaling may exacerbate metabolic and cognitive dysfunction. For instance, attenuation of estrogen and its receptors during the post-menopausal stage is linked to dyslipidemia and cognitive impairment [79]. Similarly, female mice appear partially protected from diet-induced metabolic disturbances; when exposed to a high-fat diet, males develop hyperinsulinemia, glucose intolerance, and systemic inflammation, whereas females maintain a more favorable inflammatory and metabolic profile [80].

Beyond hormonal influences, several biological differences between sexes may contribute to female resilience. Males and females differ in lipid metabolism, which may influence susceptibility to metabolic and cardiovascular disorders [19, 20]. In addition, sex-dependent differences have been consistently reported in mitochondrial redox regulation and oxidative stress responses. Experimental evidence indicates that females generally exhibit greater antioxidant capacity, including higher activity of key antioxidant enzymes and improved mitochondrial redox homeostasis compared with males. These differences are associated with reduced mitochondrial ROS production, improved respiratory efficiency, and increased resistance to oxidative damage across tissues, including the brain [81]. Collectively, these sex-dependent differences in lipid metabolism, inflammatory regulation, and mitochondrial homeostasis likely contribute to the delayed onset of hippocampal mitochondrial dysfunction observed in LDLr^−/−^ females.

A key strength of our study is the focus on middle-aged LDLr^⁻/⁻^ mice, a period that closely models the stage at which chronic hypercholesterolemia emerges as a critical risk factor for dementia in humans [9, 13]. The pronounced hippocampal bioenergetic failure we identified at this stage aligns with, and extends, a growing body of evidence indicating that middle age represents a phase of accelerating neuropathology in this model. Previously, we demonstrated that middle-aged LDLr^⁻/⁻^ mice exhibit marked antioxidant imbalance and oxidative damage in the prefrontal cortex, including elevated lipid peroxidation, disrupted glutathione metabolism, and increased acetylcholinesterase activity [35]. These signs of cellular stress are further compounded by evidence of synaptic decline, as both our group [35] and Mulder and collaborators [82] have reported decreased synaptophysin content at this age. Functionally, these molecular and structural alterations are accompanied by impaired spatial memory in the water maze and by pronounced microglial morphological changes indicative of neuroinflammation [76]. Therefore, the severe mitochondrial dysfunction observed in the present study provides a compelling mechanistic link suggesting that a collapse in energy metabolism may underpin the previously documented cascade of oxidative stress, synaptic decay, and cognitive impairment that characterizes the middle-aged hypercholesterolemic phenotype.

Finally, considering behavioral performance alongside hippocampal mitochondrial bioenergetics, we performed a correlation analysis (Supplementary Fig. 2) and identified positive associations between locomotor parameters assessed in the OF and EPM (summarized as locomotor score), hippocampal bioenergetic parameters (hippocampal bioenergetics score), and BAT mitochondrial parameters (BAT thermogenic score). Based on these integrative analyses, we found that increased cholesterol levels positively correlated with hyperlocomotion, but inversely correlated with spatial memory in the OL test. These observations align with reports that hypercholesterolemia impairs cognition and synaptic function, whereas lipid-lowering interventions, such as statins, can improve memory and reduce neuroinflammation [83, 84]. Notably, PCSK9 inhibition lowers cholesterol without affecting locomotor or cognitive performance in some models [85], suggesting that the behavioral effects of cholesterol-lowering strategies depend on experimental context. We also identified a positive correlation between hippocampal and BAT scores, suggesting that broad metabolic disruption, coupled with mitochondrial dysfunction, is associated with both central and peripheral alterations in the context of chronic hypercholesterolemia. Interestingly, our findings highlight a potential crosstalk between mitochondrial function in the hippocampus and BAT, reflecting the convergence of systemic and neural metabolism in this model. We also performed an integrative analysis aimed at identifying a “signature” of the LDLr^⁻/⁻^ genotype including both sexes (Supplementary Fig. 4). This approach revealed that plasma cholesterol levels, followed by locomotor score, triglyceride levels, time spent in the periphery of the OF, hippocampal bioenergetics score, and location index were the most informative variables for characterizing this model, reinforcing the impact of cholesterol metabolism on both CNS function and behavior.

Altogether, our findings emphasize the necessity of including sex as a critical biological variable in studies of neurodegeneration and metabolic disease. The results provide new insight into the age- and sex-dependent hippocampal vulnerability to mitochondrial dysfunction in FH and point to the convergence of systemic and neural metabolism as a key component in the onset of cognitive impairment. It is also important to consider that aging has a profound impact on females, particularly due to hormonal fluctuations and the gradual decline in estrogen levels, which can exacerbate mitochondrial dysfunction and increase neural susceptibility to metabolic and oxidative stress [81]. Therefore, interventions targeting mitochondrial health and hormonal balance may represent promising strategies for preserving brain function in dyslipidemic conditions.

## Supplementary information

Supplementary Material 1.

## Data Availability

The data that support the findings of this study are available from the first author, N. M. C. P. L. and the corresponding author, A.F.B., upon request.

## Abbreviations

AA: Antimycin A
AChE: Acetylcholinesterase
AD: Alzheimer’s Disease
ADHD: attention‑deficit/hyperactivity disorder
ADP: Adenosine Diphosphate
ANOVA: Analysis of Variance
Apo B-100: Apolipoprotein B-100
ARRIVE: Reporting of In Vivo Experiments
ASCVD: Atherosclerotic Cardiovascular Disease
ATP: Adenosine Triphosphate
Aβ: Beta-amyloid
BAT: Brown Adipose Tissue
BBB: Blood–Brain Barrier
BSA: Bovine Serum Albumin
Ca²^+^: Calcium
CCCP: Carbonyl Cyanide m-chlorophenylhydrazone
COMT: Catechol-O-methyltransferase
CoQ10: Coenzyme Q10
CVD: Cardiovascular Disease
D: Displaced Object
EPM: Elevated Plus Maze
ETS: Electron Transport System
FFAs: Free Fatty Acids
GDP: Guanosine Diphosphate
HeFH: Heterozygous Familial Hypercholesterolemia
HMG-CoA Reductase: 3-hydroxy-3-methylglutaryl-coenzyme A reductase
HoFH: Homozygous Familial Hypercholesterolemia
HRR: High-Resolution Respirometry
i.p.: Intraperitoneal, IMM: Inner Mitochondrial Membrane
LDL: Low-Density Lipoprotein
LDL-C: Circulating Low-Density Lipoprotein
LDLr: LDL Receptor knockout mice
LI: Location Index, M:Malate
MCI: Mild Cognitive Impairment (for CCL)
MWM: Morris Water Maze
NADPH: Nicotinamide Adenine Dinucleotide Phosphate (Reduced)
ND: Non-Displaced Object
NIH: National Institutes of Health
O₂: Oxygen
OF: Open Field
OL: Object Location
OMY: Oligomycin
OXPHOS: Oxidative Phosphorylation
P: Pyruvate
PCSK9: Proprotein Convertase Subtilisin/Kexin Type 9
PM: Pyruvate and Malate
RB: Reaction Buffer
ROS: Reactive Oxygen Species
ROT: Rotenone, S:Succinate
SOD: Superoxide Dismutase
TCA: Tricarboxylic Acid Cycle
UCL: University College London
UCP-1: Uncoupling Protein 1
VLDL: Very-Low-Density Lipoprotein
WAT: White Adipose Tissue
WT: Wild-Type

## References

[1] Alves RJ, Takao Suehiro Junior A, Brailowsky Pellegrino L. Hipercolesterolemia familiar homozigótica e heterozigótica grave: epidemiologia, diagnóstico e tratamento. Revista Da Sociedade de Cardiologia Do Estado de São Paulo. 2021;31(1):14–22. 10.29381/0103-8559/2021310114-22.

[2] Defesche JC, Gidding SS, Harada-Shiba M, Hegele RA, Santos RD, Wierzbicki AS. Familial hypercholesterolaemia. Nat Reviews Disease Primers. 2017;3(1). 10.1038/nrdp.2017.93. 10.1038/nrdp.2017.93 29219151

[3] Goldstein JL, Brown MS. The low-density lipoprotein pathway and its relation to atherosclerosis. Annu Rev Biochem. 1977. 10.1146/annurev.bi.46.070177.004341. 197883 10.1146/annurev.bi.46.070177.004341

[4] Hobbs HH, Brown MS, Goldstein JL. Molecular genetics of the LDL receptor gene in familial hypercholesterolemia. Hum Mutat. 1992;1(6):445–66. 10.1002/humu.1380010602;wgroup:string:publication. 1301956 10.1002/humu.1380010602

[5] Zambón D, Quintana M, Mata P, Alonso R, Benavent J, Cruz-Sánchez F, et al. Higher incidence of mild cognitive impairment in familial hypercholesterolemia. Am J Med. 2010;123(3):267–74. 10.1016/j.amjmed.2009.08.015. 20193836 10.1016/j.amjmed.2009.08.015 PMC2844655

[6] Basak JM, Verghese PB, Yoon H, Kim J, Holtzman DM. Low-density lipoprotein receptor represents an apolipoprotein E-independent pathway of Aβ uptake and degradation by astrocytes. J Biol Chem. 2012;287(17):13959–71. 10.1074/jbc.m111.288746/attachment/9c3a52a5-60f2-4c95-9ab7-7aee307505a8/mmc1.pdf. 22383525 10.1074/jbc.M111.288746 PMC3340151

[7] Castellano JM, Deane R, Gottesdiener AJ, Verghese PB, Stewart FR, West T, et al. Low-density lipoprotein receptor overexpression enhances the rate of brain-to-blood Aβ clearance in a mouse model of β-amyloidosis. Proc Natl Acad Sci USA. 2012;109(38):15502–7. 10.1073/pnas.1206446109/suppl_file/pnas.201206446si.pdf. 22927427 10.1073/pnas.1206446109 PMC3458349

[8] Shi Y, Andhey PS, Ising C, Wang K, Snipes LL, Boyer K, et al. Overexpressing low-density lipoprotein receptor reduces tau-associated neurodegeneration in relation to apoE-linked mechanisms. Neuron. 2021;109(15):2413–e24267. 10.1016/j.neuron.2021.05.034. 34157306 10.1016/j.neuron.2021.05.034 PMC8349883

[9] Livingston G, Huntley J, Liu KY, Costafreda SG, Selbæk G, Alladi S, et al. Dementia prevention, intervention, and care: 2024 report of the Lancet standing Commission. Lancet. 2024;404(10452):572–628. 10.1016/S0140-6736(24)01296-0. 39096926 10.1016/S0140-6736(24)01296-0

[10] Solomon A, Kivipelto M, Wolozin B, Zhou J, Whitmer RA. Midlife serum cholesterol and increased risk of Alzheimer’s and vascular dementia three decades later. Dement Geriatr Cogn Disord. 2009;28(1):75–80. 10.1159/000231980. 19648749 10.1159/000231980 PMC2814023

[11] Kivipelto M, Helkala EL, Laakso MP, Hänninen T, Hallikainen M, Alhainen K, et al. Midlife vascular risk factors and Alzheimer’s disease in later life: longitudinal, population-based study. BMJ: Br Med J. 2001;322(7300):1447. 10.1136/bmj.322.7300.1447. 11408299 10.1136/bmj.322.7300.1447 PMC32306

[12] Ariza M, Cuenca N, Mauri M, Jurado MA, Garolera M. Neuropsychological performance of young familial hypercholesterolemia patients. Eur J Intern Medicine: e. 2016;29–31. 10.1016/j.ejim.2016.05.009. 10.1016/j.ejim.2016.05.009 27264249

[13] Scilletta S, Miano N, Di Marco M, Musmeci M, Carasi S, Capuccio S, et al. Evaluation of cognitive profile and subclinical vascular damage in subjects with genetically confirmed familial hypercholesterolemia. Eur J Intern Med. 2026;143:106511. 10.1016/j.ejim.2025.106511. 40957754 10.1016/j.ejim.2025.106511

[14] Ferreira NV, Bittencourt MS, Generoso G, Gomes-Gonçalves N, Barreto SM, Giatti L, et al. Non-linear associations of serum lipid levels with cognitive decline: Findings from the ELSA-Brasil cohort. Atherosclerosis. 2025;403. 10.1016/j.atherosclerosis.2025.119159. 10.1016/j.atherosclerosis.2025.119159 40043444

[15] Coutinho ER, Miname MH, Rocha VZ, Bittencourt MS, Jannes CE, Tada MT, et al. Familial hypercholesterolemia and cardiovascular disease in older individuals. Atherosclerosis. 2021;318:32–7. 10.1016/j.atherosclerosis.2020.12.012. 33450476 10.1016/j.atherosclerosis.2020.12.012

[16] Ito MK, Watts GF. Challenges in the Diagnosis and Treatment of Homozygous Familial Hypercholesterolemia. Drugs. 2015;75(15):1715–24. 10.1007/s40265-015-0466-y. 26370207 10.1007/s40265-015-0466-y PMC4611011

[17] Iyen B, Qureshi N, Weng S, Roderick P, Kai J, Capps N, et al. Sex differences in cardiovascular morbidity associated with familial hypercholesterolemia: A retrospective cohort study of the UK Simon Broome register linked to national hospital records. Atherosclerosis. 2020;315:131–7. 10.1016/j.atherosclerosis.2020.10.895. 33187671 10.1016/j.atherosclerosis.2020.10.895 PMC7754706

[18] Pajak A, Szafraniec K, Polak M, Drygas W, Piotrowski W, Zdrojewski T, et al. Prevalence of familial hypercholesterolemia: A meta-analysis of six large, observational, population-based studies in Poland. Archives Med Sci. 2016;687–96. 10.5114/aoms.2016.59700. 10.5114/aoms.2016.59700 PMC4947614 27478447

[19] Santosa S, Jensen MD. The sexual dimorphism of lipid kinetics in humans. Front Endocrinol. 2015. 10.3389/fendo.2015.00103. 10.3389/fendo.2015.00103 PMC4489151 26191040

[20] Lorbek G, Perše M, Horvat S, Björkhem I, Rozman D. Sex differences in the hepatic cholesterol sensing mechanisms in mice. Molecules. 2013;18(9):11067–85. 10.3390/molecules180911067. 24025456 10.3390/molecules180911067 PMC6270450

[21] De Felice FG, Lourenco MV, Ferreira ST. How does brain insulin resistance develop in Alzheimer’s disease? Alzheimer’s Dement. 2014;10(1):S26–32. 10.1016/j.jalz.2013.12.004. 24529521 10.1016/j.jalz.2013.12.004

[22] Kothari A, McCutcheon C, Graham ID. Defining Integrated Knowledge Translation and Moving Forward: A Response to Recent Commentaries. Int J Health Policy Manage. 2017;6(5):299. 10.15171/ijhpm.2017.15. 10.15171/ijhpm.2017.15 PMC5417154 28812820

[23] Bharath LP, Agrawal M, McCambridge G, Nicholas DA, Hasturk H, Liu J, et al. Metformin Enhances Autophagy and Normalizes Mitochondrial Function to Alleviate Aging-Associated Inflammation. Cell Metabol. 2020;32(1):44–e556. 10.1016/j.cmet.2020.04.015. 10.1016/j.cmet.2020.04.015 PMC7217133 32402267

[24] Guo J, Huang X, Dou L, Yan M, Shen T, Tang W, et al. Aging and aging-related diseases: from molecular mechanisms to interventions and treatments. Signal Transduct Target Therapy 2022. 2022;7(1):1. 10.1038/s41392-022-01251-0. 10.1038/s41392-022-01251-0 PMC9755275 36522308

[25] López-Otín C, Blasco MA, Partridge L, Serrano M, Kroemer G. Hallm Aging Cell. 2013;153(6):1194–217. 10.1016/j.cell.2013.05.039. 10.1016/j.cell.2013.05.039 PMC3836174 23746838

[26] Van der Laan L, Cardenas A, Vermeulen R, Fadadu RP, Hubbard AE, Phillips RV, et al. Epigenetic aging biomarkers and occupational exposure to benzene, trichloroethylene and formaldehyde. Environ Int. 2022;158:106871. 10.1016/j.envint.2021.106871. 34560324 10.1016/j.envint.2021.106871 PMC9084243

[27] Ishibashi S, Brown MS, Goldstein JL, Gerard RD, Hammer RE, Herz J. 1993. Hypercholesterolemia in Low Density Lipoprotein Receptor Knockout Mice and its Reversal by Adenovirus-mediated Gene Delivery. 10.1172/jci116663 10.1172/JCI116663 PMC294927 8349823

[28] de Oliveira J, Hort MA, Moreira ELG, Glaser V, Ribeiro-do-Valle RM, Prediger RD, et al. Positive correlation between elevated plasma cholesterol levels and cognitive impairments in LDL receptor knockout mice: Relevance of cortico-cerebral mitochondrial dysfunction and oxidative stress. Neuroscience. 2011;197:99–106. 10.1016/j.neuroscience.2011.09.009. 21945034 10.1016/j.neuroscience.2011.09.009

[29] De Oliveira J, Moreira ELG, Dos Santos DB, Piermartiri TC, Dutra RC, Pinton S, et al. Increased susceptibility to amyloid-β-induced neurotoxicity in mice lacking the low-density lipoprotein receptor. J Alzheimer’s Disease. 2014;41(1):43–60. 10.3233/JAD-132228. 24577472 10.3233/JAD-132228

[30] de Paula GC, Brunetta HS, Engel DF, Gaspar JM, Velloso LA, Engblom D, et al. Hippocampal Function Is Impaired by a Short-Term High-Fat Diet in Mice: Increased Blood–Brain Barrier Permeability and Neuroinflammation as Triggering Events. Front NeuroSci. 2021;15. 10.3389/fnins.2021.734158. 10.3389/fnins.2021.734158 PMC8600238 34803583

[31] Moreira ELG, De Oliveira J, Nunes JC, Santos DB, Nunes FC, Vieira DSC, et al. Age-Related cognitive decline in hypercholesterolemic LDL receptor knockout mice (LDLr^–/–^): Evidence of antioxidant imbalance and increased acetylcholinesterase activity in the prefrontal cortex. J Alzheimer’s Disease. 2012;32(2):495–511. 10.3233/JAD-2012-120541. 22810096 10.3233/JAD-2012-120541

[32] de Oliveira J, Moreira ELG, de Bem AF. Beyond cardiovascular risk: Implications of Familial hypercholesterolemia on cognition and brain function. Ageing Res Rev. 2024. 10.1016/j.arr.2023.102149. 38056504 10.1016/j.arr.2023.102149

[33] Szczepanik JC, de Oliveira PA, de Oliveira J, Mack JM, Engel DF, Rial D, et al. Caffeine Mitigates the Locomotor Hyperactivity in Middle-aged Low‐density Lipoprotein Receptor (LDLr)‐Knockout Mice. CNS Neurosci Ther. 2016;22(5):420. 10.1111/cns.12544. 27012312 10.1111/cns.12544 PMC6492875

[34] Engel DF, de Oliveira J, Lopes JB, Santos DB, Moreira ELG, Farina M, et al. Is there an association between hypercholesterolemia and depression? Behavioral evidence from the LDLr-/- mouse experimental model. Behav Brain Res. 2016;311:31–8. 10.1016/j.bbr.2016.05.029. 27185735 10.1016/j.bbr.2016.05.029

[35] Rodrigues MS, do Nascimento NB, Farias HR, Schons T, Machado AG, Behenck E, et al. Microglia contribute to cognitive decline in hypercholesterolemic LDLr–/– mice. J Neurochem. 2023;168(8):1565–86. 10.1111/jnc.15952. 37694813 10.1111/jnc.15952

[36] Wang SH, Huang Y, Yuan Y, Xia WQ, Wang P, Huang R. LDL receptor knock-out mice show impaired spatial cognition with hippocampal vulnerability to apoptosis and deficits in synapses. Lipids Health Dis. 2014;13(1):1–11. 10.1186/1476-511x-13-175. 10.1186/1476-511X-13-175 PMC4258039 25413784

[37] De Oliveira J, Engel DF, De Paula GC, Dos Santos DB, Lopes JB, Farina M, et al. High Cholesterol Diet Exacerbates Blood-Brain Barrier Disruption in LDLr-/- Mice: Impact on Cognitive Function. J Alzheimer’s Disease. 2020;78(1):97–115. 10.3233/jad-200541. 32925052 10.3233/JAD-200541 PMC7683087

[38] Suárez-Rivero JM, de la Mata M, Pavón AD, Villanueva-Paz M, Povea-Cabello S, Cotán D, et al. Intracellular cholesterol accumulation and coenzyme Q10 deficiency in Familial Hypercholesterolemia. Biochim et Biophys Acta - Mol Basis Disease. 2018;1864(12):3697–713. 10.1016/j.bbadis.2018.10.009. 10.1016/j.bbadis.2018.10.009 30292637

[39] Ruiz-Pesini E, Bayona-Bafaluy MP, Sanclemente T, Puzo J, Montoya J, Pacheu-Grau D. Mitochondrial Genetic Background May Impact Statins Side Effects and Atherosclerosis Development in Familial Hypercholesterolemia. Int J Mol Sci. 2023;24(1). 10.3390/ijms24010471. 10.3390/ijms24010471 PMC9820128 36613915

[40] Lee JH, Park A, Oh KJ, Lee SC, Kim WK, Bae KH. The Role of Adipose Tissue Mitochondria: Regulation of Mitochondrial Function for the Treatment of Metabolic Diseases. Int J Mol Sci 2019. 2019;20(19):4924. 10.3390/ijms20194924. 10.3390/ijms20194924 PMC6801758 31590292

[41] Zhang Y, Wong HS. Are mitochondria the main contributor of reactive oxygen species in cells. J Exp Biol. 2021;224(5). 10.1242/jeb.221606/237509. 10.1242/jeb.221606 33707189

[42] Bournat JC, Brown CW. Mitochondrial Dysfunction in Obesity. Doi: 10.1097/MED; 2010. 10.1097/MED.0b013e32833c3026 PMC5001554 20585248

[43] Victor V, Rocha M, Sola E, Banuls C, Garcia-Malpartida K, Hernandez- Mijares A. Oxidative Stress, Endothelial Dysfunction and Atherosclerosis. Curr Pharm Design. 2009;15(26):2988–3002. 10.2174/138161209789058093. 10.2174/138161209789058093 19754375

[44] Cinti S. Transdifferentiation properties of adipocytes in the adipose organ. Am J Physiol - Endocrinol Metabolism. 2009;297(5). 10.1152/AJPENDO.00183.2009. 10.1152/ajpendo.00183.2009 19458063

[45] Vamecq J, Dessein A-F, Fontaine M, Briand G, Porchet N, Latruffe N et al. 2012. Mitochondrial Dysfunction and Lipid Homeostasis. vol. 13. 10.2174/138920012803762792 22978394

[46] Prut L, Belzung C. The open field as a paradigm to measure the effects of drugs on anxiety-like behaviors: A review. Eur J Pharmacol. 2003;463(1–3):3–33. 10.1016/s0014-2999(03)01272-x. 12600700 10.1016/s0014-2999(03)01272-x

[47] Assini FL, Duzzioni M, Takahashi RN. Object location memory in mice: Pharmacological validation and further evidence of hippocampal CA1 participation. Behav Brain Res. 2009;204(1):206–11. 10.1016/j.bbr.2009.06.005. 19523494 10.1016/j.bbr.2009.06.005

[48] Murai T, Okuda S, Tanaka T, Ohta H. Characteristics of object location memory in mice: Behavioral and pharmacological studies. Physiol Behav. 2007;90(1):116–24. 10.1016/j.physbeh.2006.09.013. 17049363 10.1016/j.physbeh.2006.09.013

[49] Rodgers RJ, Dalvi A. Anxiety, defence and the elevated plus-maze. Neurosci Biobehavioral Reviews. 1997;21(6):801–10. 10.1016/s0149-7634(96)00058-9. 10.1016/s0149-7634(96)00058-9 9415905

[50] Vilela WR, Bellozi PMQ, Picolo VL, Cavadas BN, Marques KVS, Pereira LTG, et al. Early-life metabolic dysfunction impairs cognition and mitochondrial function in mice. J Nutr Biochem. 2023;117:109352. 10.1016/j.jnutbio.2023.109352. 37061011 10.1016/j.jnutbio.2023.109352

[51] Ishibashi S, Goldstein JL, Brown MS, Herz J, Burns DK. Massive xanthomatosis and atherosclerosis in cholesterol-fed low density lipoprotein receptor-negative mice. J Clin Invest. 1994;93(5):1885–93. 10.1172/jci117179. 8182121 10.1172/JCI117179 PMC294295

[52] Marsh MM, Walker VR, Curtiss LK, Banka CL. Protection against atherosclerosis by estrogen is independent of plasma cholesterol levels in LDL receptor-deficient mice. J Lipid Res. 1999;40(5):893–900. 10.1016/s0022-2275(20)32124-6. 10224158

[53] Ghosh SS, Wang J, Yannie PJ, Sandhu YK, Korzun WJ, Ghosh S. Dietary supplementation with galactooligosaccharides attenuates high-fat, high-cholesterol diet-induced glucose intolerance and disruption of colonic mucin layer in C57BL/6 mice and reduces atherosclerosis in LDLr -/-mice. J Nutr. 2020;150(2):285–93. 10.1093/jn/nxz233. 31586202 10.1093/jn/nxz233

[54] Rinninger F, Heine M, Singaraja R, Hayden M, Brundert M, Ramakrishnan R, et al. High density lipoprotein metabolism in low density lipoprotein receptor-deficient mice. J Lipid Res. 2014;55(9):1914–24. 10.1194/jlr.M048819. 24954421 10.1194/jlr.M048819 PMC4617360

[55] Rudling M, Angelin BO. Growth hormone reduces plasma cholesterol in LDL receptor-deficient mice. Meta, 2001. 10.1096/fj.00-0715com. 10.1096/fj.00-0715com 11387232

[56] Ferreira DF, Fiamoncini J, Prist IH, Ariga SK, De Souza HP, De Lima TM. Novel role of TLR4 in NAFLD development: Modulation of metabolic enzymes expression. Biochimica et Biophysica Acta (BBA) -. Mol Cell Biology Lipids. 2015;1851(10):1353–9. 10.1016/j.bbalip.2015.07.002. 10.1016/j.bbalip.2015.07.002 26172853

[57] Sithu SD, Malovichko MV, Riggs KA, Wickramasinghe NS, Winner MG, Agarwal A, et al. Atherogenesis and metabolic dysregulation in LDL receptor–knockout rats. JCI Insight. 2017;2(9). 10.1172/jci.insight.86442. 10.1172/jci.insight.86442 PMC5414561 28469073

[58] Li Q, Wang O, Ji B, Zhao L, Zhao L. 2023. Alcohol, White Adipose Tissue, and Brown Adipose Tissue: Mechanistic Links to Lipogenesis and Lipolysis. Nutrients 2023, Vol. 15, Page 2953 15(13): 2953. 10.3390/nu15132953 10.3390/nu15132953 PMC10346806 37447280

[59] Ying Z, Tramper N, Zhou E, Boon MR, Rensen PCN, Kooijman S. Role of thermogenic adipose tissue in lipid metabolism and atherosclerotic cardiovascular disease: Lessons from studies in mice and humans. Cardiovascular Res. 2023;905–18. 10.1093/cvr/cvac131. 10.1093/cvr/cvac131 PMC10153643 35944189

[60] Klingenberg M, Huang S-G. 1999. Structure and function of the uncoupling protein from brown adipose tissue. Biochimica et Biophysica Acta (BBA)-Biomembranes, 10.1016/S0005-2736(98)00232-6 10.1016/s0005-2736(98)00232-6 9889383

[61] Liu J, Li J, Li WJ, Wang CM. The role of uncoupling proteins in diabetes mellitus. J Diabetes Res. 2013. 10.1155/2013/585897. 23841103 10.1155/2013/585897 PMC3687498

[62] Worthmann A, Schlein C, Berbée JFP, Rensen PCN, Heeren J, Bartelt A. Effects of pharmacological thermogenic adipocyte activation on metabolism and atherosclerotic plaque regression. Nutrients. 2019;11(2). 10.3390/nu11020463. 10.3390/nu11020463 PMC6412269 30813320

[63] Liu L, Liang C, Wang X, Ding X, Lu Y, Dong J, et al. Surgical fat removal exacerbates metabolic disorders but not atherogenesis in LDLR–/– mice fed on high-fat diet. Sci Rep. 2019;9(1). 10.1038/s41598-019-54392-8. 10.1038/s41598-019-54392-8 PMC6883051 31780791

[64] Elder GA, Ragnauth A, Dorr N, Franciosi S, Schmeidler J, Haroutunian V, et al. Increased locomotor activity in mice lacking the low-density lipoprotein receptor. Behav Brain Res. 2008;191(2):256. 10.1016/j.bbr.2008.03.036. 18466986 10.1016/j.bbr.2008.03.036 PMC4662864

[65] Qian Y, Lei G, Castellanos FX, Forssberg H, Heijtz RD. Deficits in fine motor skills in a genetic animal model of ADHD. Behav Brain Funct 2010. 2010;6(1 6):51. 10.1186/1744-9081-6-51. 10.1186/1744-9081-6-51 PMC2940855 20809977

[66] Corona JC. Rodent research of attention-deficit/hyperactivity disorder: insights into widely used animal models. Lab Anim Res 2025. 2025;41(1):1. 10.1186/s42826-025-00255-5. 10.1186/s42826-025-00255-5 PMC12455772 40988072

[67] Mejias R, Rodriguez-Gotor JJ, Niwa M, Krasnova IN, Adamczyk A, Han M, et al. Increased novelty-induced locomotion, sensitivity to amphetamine, and extracellular dopamine in striatum of Zdhhc15-deficient mice. Translational Psychiatry. 2021;11(1):65. 10.1038/s41398-020-01194-6. 33462194 10.1038/s41398-020-01194-6 PMC7813841

[68] Pinho CM, Olescowicz G, Bevilacqua LM, de Amorim GES, Platt N, da Silva Räder MA, et al. Sex Differences on Social and Anxiety-Related Responses in Low-Density Lipoprotein Receptor Knockout Mice. Mol Neurobiol 2026. 2026;63(1 63):472. 10.1007/s12035-026-05742-x. 10.1007/s12035-026-05742-x PMC12946358 41748982

[69] De Oliveira J, Moreira ELG, Mancini G, Hort MA, Latini A, Ribeiro-Do-Valle RM, et al. Diphenyl diselenide prevents cortico-cerebral mitochondrial dysfunction and oxidative stress induced by hypercholesterolemia in LDL receptor knockout mice. Neurochem Res. 2013;38(10):2028–36. 10.1007/s11064-013-1110-4. 23881289 10.1007/s11064-013-1110-4

[70] Lopes JB, de Oliveira J, Engel DF, de Paula GC, Moreira ELG, de Bem AF. Efficacy of Donepezil for Cognitive Impairments in Familial Hypercholesterolemia: Preclinical Proof of Concept. CNS Neurosci Ther. 2015;21(12):964. 10.1111/CNS.12479. 26555675 10.1111/cns.12479 PMC6493049

[71] Zelko MD, Robinson SR, Hill-Yardin EL, Nasser H. Resolving anxiety-like behaviour inconsistencies in the elevated plus maze by tracking exploration depth and timing. Behav Res Methods 2025. 2025;57(8):8. 10.3758/s13428-025-02738-8. 10.3758/s13428-025-02738-8 PMC12209025 40587031

[72] Walf AA, Frye CA. The use of the elevated plus maze as an assay of anxiety-related behavior in rodents. Nat Protoc. 2007;2(2):322. 10.1038/nprot.2007.44. 17406592 10.1038/nprot.2007.44 PMC3623971

[73] Wang T, Chen Y, Zou Y, Pang Y, He X, Chen Y, et al. Locomotor Hyperactivity in the Early-Stage Alzheimer’s Disease-like Pathology of APP/PS1 Mice: Associated with Impaired Polarization of Astrocyte Aquaporin 4. Aging Disease. 2022;13(5):1504. 10.14336/ad.2022.0219. 36186142 10.14336/AD.2022.0219 PMC9466968

[74] Zou L, Tian Y, Wang Y, Chen D, Lu X, Zeng Z, et al. High-cholesterol diet promotes depression- and anxiety-like behaviors in mice by impact gut microbe and neuroinflammation. J Affect Disord. 2023;327:425–38. 10.1016/j.jad.2023.01.122. 36738999 10.1016/j.jad.2023.01.122

[75] Squire LR. Memory and the Hippocampus: A Synthesis From Findings With Rats, Monkeys, and Humans. Volume 99. Psychol Rev; 1992. 10.1037/0033-295x.99.2.195. 10.1037/0033-295x.99.2.195 1594723

[76] De Oliveira J, Engel DF, De Paula GC, Melo HM, Lopes SC, Ribeiro CT, et al. LDL Receptor Deficiency Does not Alter Brain Amyloid-β Levels but Causes an Exacerbation of Apoptosis. J Alzheimer’s Disease: JAD. 2020;73(2):585–96. 10.3233/jad-190742. 31815695 10.3233/JAD-190742

[77] do Nascimento NB, Farias HR, Schons T, Padilha APZ, Costa MV, Peres AM, et al. Metformin Ameliorates Cognitive Deficits and Neuroinflammation in a Mouse Model of Familial Hypercholesterolemia. Neurochemical Res 2026. 2026;51(1 51):60. 10.1007/s11064-025-04658-7. 10.1007/s11064-025-04658-7 PMC12830493 41578026

[78] Liu L, Zhang C, Lv X, Lai X, Xu L, Feng J, et al. Sex-specific associations between lipids and cognitive decline in the middle-aged and elderly: a cohort study of Chinese adults. Alzheimer’s Res Therapy. 2020;12(1). 10.1186/s13195-020-00731-1. 10.1186/s13195-020-00731-1 PMC7722300 33287901

[79] Meng Q, Chao Y, Zhang S, Ding X, Feng H, Zhang C, et al. Attenuation of estrogen and its receptors in the post-menopausal stage exacerbates dyslipidemia and leads to cognitive impairment. Mol Brain. 2023;16(1). 10.1186/s13041-023-01068-0. 10.1186/s13041-023-01068-0 PMC10662842 37986006

[80] Pettersson US, Waldén TB, Carlsson PO, Jansson L, Phillipson M. Female Mice are Protected against High-Fat Diet Induced Metabolic Syndrome and Increase the Regulatory T Cell Population in Adipose Tissue. PLoS ONE. 2012;7(9). 10.1371/journal.pone.0046057. 10.1371/journal.pone.0046057 PMC3458106 23049932

[81] Lejri I, Grimm A, Eckert A. Mitochondria, Estrogen and Female Brain Aging. Front Aging Neurosci. 2018;10(APR):124. 10.3389/fnagi.2018.00124. 29755342 10.3389/fnagi.2018.00124 PMC5934418

[82] Mulder M, Koopmans G, Wassink G, Mansouri G, Al., Simard ML, Havekes LM, et al. LDL receptor deficiency results in decreased cell proliferation and presynaptic bouton density in the murine hippocampus. Neurosci Res. 2007;59(3):251–6. 10.1016/j.neures.2007.07.004. 17720268 10.1016/j.neures.2007.07.004

[83] Tong XK, Trigiani LJ, Hamel E. High cholesterol triggers white matter alterations and cognitive deficits in a mouse model of cerebrovascular disease: benefits of simvastatin. Cell Death Dis. 2019;10(2):89. 10.1038/s41419-018-1199-0. 30692517 10.1038/s41419-018-1199-0 PMC6349936

[84] Don-Doncow N, Vanherle L, Matthes F, Petersen SK, Matuskova H, Rattik S, et al. Simvastatin therapy attenuates memory deficits that associate with brain monocyte infiltration in chronic hypercholesterolemia. NPJ Aging Mech Disease. 2021;7(1):19. 10.1038/s41514-021-00071-w. 10.1038/s41514-021-00071-w PMC8338939 34349106

[85] Schlunk F, Fischer P, Princen HMG, Rex A, Prinz V, Foddis M, et al. No effects of PCSK9-inhibitor treatment on spatial learning, locomotor activity, and novel object recognition in mice. Behav Brain Res. 2021;396. 10.1016/j.bbr.2020.112875. 10.1016/j.bbr.2020.112875 32858115

