# Supplementary material for: Sex- and age-dependent mitochondrial dysfunction and cognitive impairment in a mouse model of familial hypercholesterolemia
Source: Biol Sex Differ. 2026 Apr 10;17:109. doi: 10.1186/s13293-026-00893-x (PMC13182057; doi:10.1186/s13293-026-00893-x)
Supplement: Supplementary file 1 — Supplementary Material 1. [file 13293_2026_893_MOESM1_ESM.docx]

**APPENDIX A. SUPPLEMENTARY DATA**

**Supplementary Fig. 1**

**
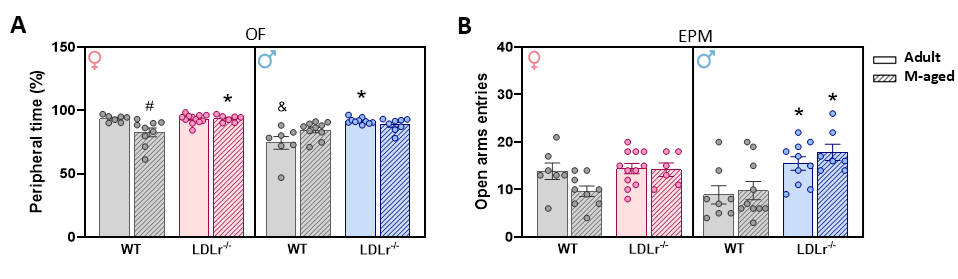
**

**Supplementary Figure 1. Sex-specific differences in behavioral parameters.** (A) Time spent in the periphery zone (%) was assessed in the OF arena for 5 minutes, and (B) the number of entries into the open arms in EPM were measured to evaluate behavioral parameters. n= 6-12/group. All data were expressed as mean ± SEM. Statistical analysis was performed using a Three-way ANOVA to include sex, age and genotype variables. *Genotype effect (WT vs. LDLr^-/-^), ^#^Age effect (WT vs. WT, and LDLr^-/-^ vs. LDLr^-/-^), ^&^Sex effect (Male vs. Female of the same age and genotype), p< 0.05.

**Supplementary Fig. 2**

**
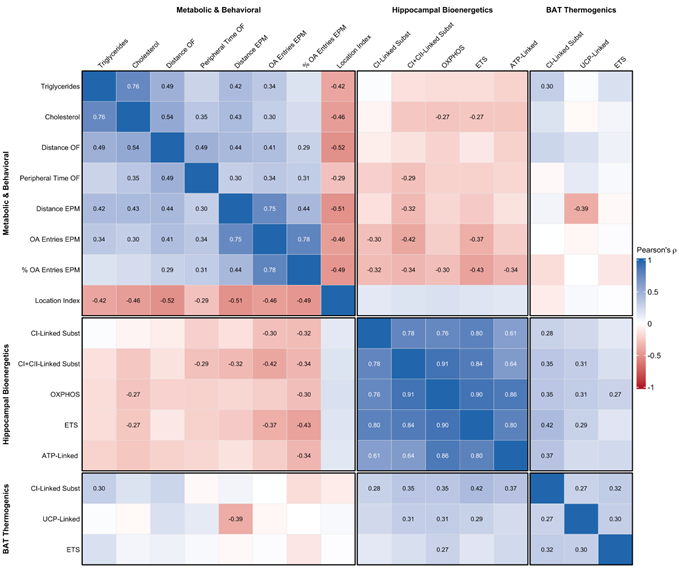
**

**Supplementary Figure 2. Comprehensive correlation matrix of metabolic, behavioral, and bioenergetic variables.** Heatmap displaying Pearson’s correlation coefficients (ρ) for all pairwise comparisons. The color scale indicates the direction and strength of the correlation, with dark blue representing strong positive correlations (ρ = 1) and dark red representing strong negative correlations (ρ = -1). Numerical values are shown exclusively for correlations with statistical significance (p<0.05). Variables were clustered a priori into three functional blocks (Metabolic & Behavioral, Hippocampal Bioenergetics, BAT Thermogenics) to facilitate the interpretation of intra- and inter-domain relationships.

**Supplementary Fig. 3**

**
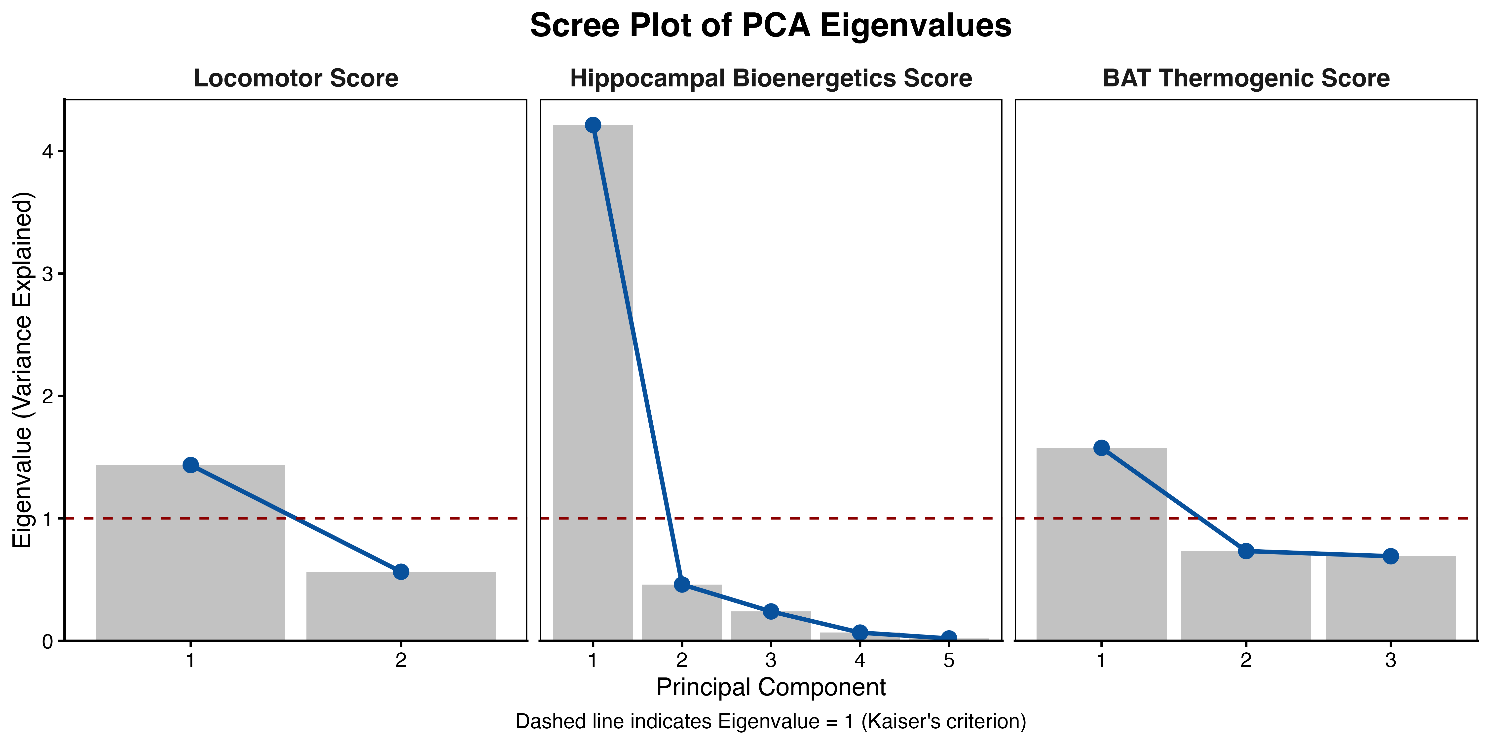
**

**Supplementary Figure 3. Scree plots validating the generation of composite scores via Principal Component Analysis.** The plots display the eigenvalues for each principal component derived from the PCA of (A) Locomotor, (B) Hippocampal Bioenergetics, and (C) BAT Thermogenic variable sets. The eigenvalue (y-axis) represents the amount of variance explained by each component. The dashed horizontal line indicates an eigenvalue of 1 (Kaiser's criterion). For all three domains, only the first principal component (PC1) had an eigenvalue substantially greater than one, explaining 71.8%, 84.3%, and 52.5% of the variance in their respective datasets and justifying their use as single composite scores.

**Supplementary Fig. 4**

**
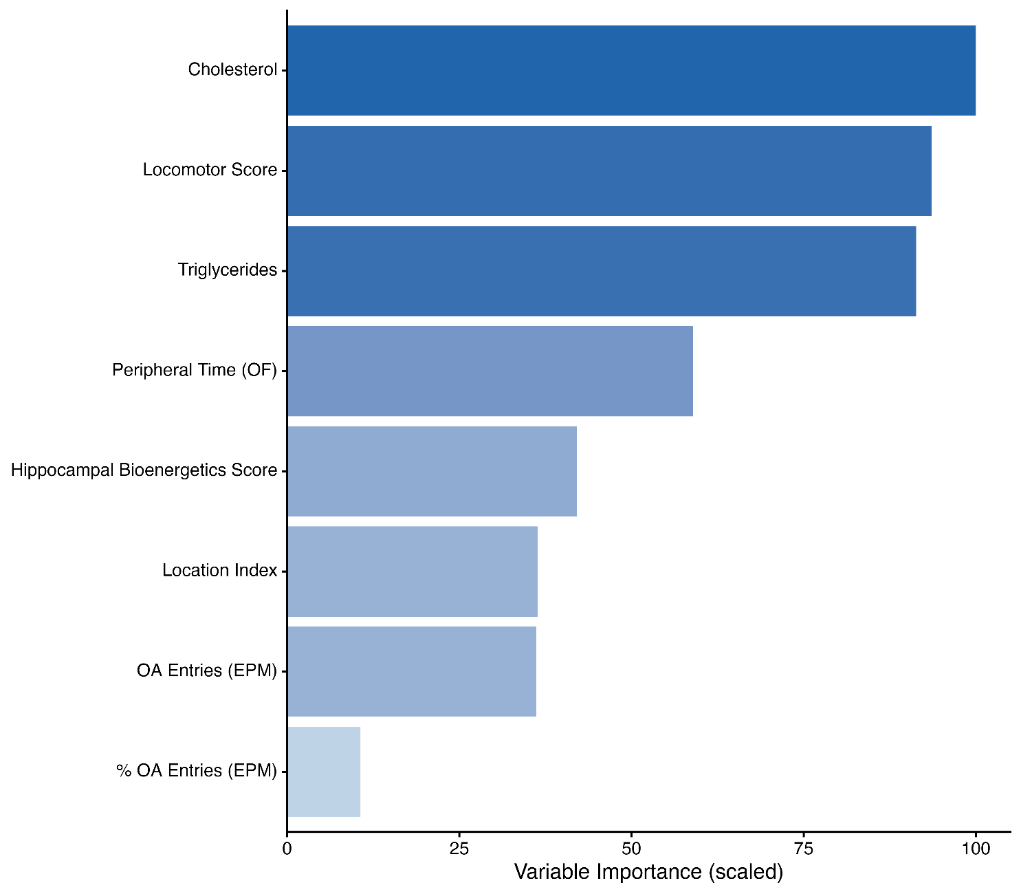
**

**Supplementary Figure 4. Variable Importance in Projection (VIP) scores from the PLS-DA model.** The bar plot shows the scaled importance of the top phenotypic variables used by the Partial Least Squares Discriminant Analysis (PLS-DA) model to classify animals by genotype. A higher VIP score indicates a greater contribution to the model's discriminatory power. Serum cholesterol, the composite locomotor score, and serum triglycerides emerged as the three most influential variables for predicting genotype, identifying dyslipidemia and hyperactivity as the core signature of the LDLr^⁻/⁻^ model.

**Supplementary Figure 5.**

**
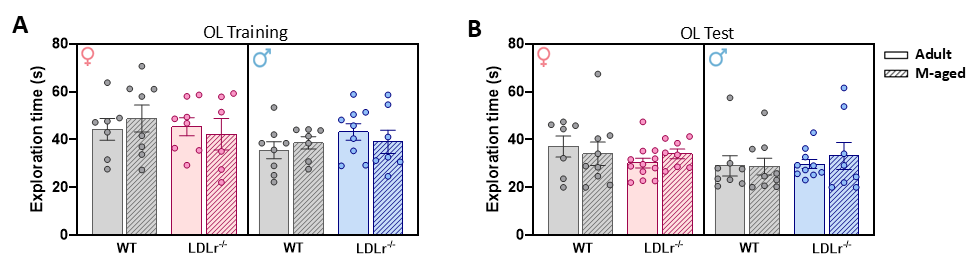
**

**Supplementary Figure 5. Total exploration time in the Object Location (OL) test.** Total exploration time (in seconds) during (A**)** OL training and (B) OL test in five minutes in each session. n= 6-12/group.
